# Mapping data stewardship in Italy: Findings from the first national survey

**DOI:** 10.12688/openreseurope.22183.1

**Published:** 2026-02-13

**Authors:** Giulia Caldoni, Vittorio Iacovella, Emma Lazzeri, Liise Lehtsalu, Francesca Marchegiani, Mauro Paschetta, Valentina Pasquale, Giulia Pedonese, Andrea Tarallo

**Affiliations:** 1 Research Division (ARIC), Alma Mater Studiorum - Università di Bologna, Bologna, Emilia-Romagna, 40126, Italy; 2 Universita degli Studi di Trento Centro Interdipartimentale Mente/Cervello, Rovereto, Trentino-Alto Adige/South Tyrol, 38068, Italy; 3 CNR - Ufficio Agenda Digitale e Processi, Rome, Lazio, 00185, Italy; 4 RDA Professionalising Data Stewardship Interest Group and RDA Data Steward Career Tracks Working Group, Brunico, Trentino Alto-Adige, 39031, Italy; 5 INFN - Laboratori Nazionali del Gran Sasso, Assergi, Abruzzo, 67100, Italy; 6 Politecnico di Torino, Turin, Piedmont, 10129, Italy; 7 Istituto Italiano di Tecnologia, Genoa, Liguria, 16163, Italy; 8 CNR - Istituto di Linguistica Computazionale "Antonio Zampolli", Pisa, Tuscany, 56124, Italy; 9 CNR - Istituto di Ricerca sugli Ecosistemi Terrestri, Lecce, Puglia, 73100, Italy

**Keywords:** survey, data stewardship, Italy, data steward, professional profile, mapping

## Abstract

Research data management plays a key role in ensuring research reproducibility and transparency by supporting data sharing in line with Open Science principles. Professional figures supporting data management practices, such as data stewards, are emerging in the international landscape, following different paths. The Italian Computing and Data Infrastructure, with the support of the Skills4EOSC project, conducted a national survey between April and December 2023 to map the presence, roles, and activities of data stewards or equivalent professionals across universities and research institutions, to provide an initial and systematic overview of the national landscape, which is known to be fragmented and lacking of clear professional recognition for research support professionals. The survey, administered in Italian through the EUSurvey platform, consisted of 13 closed-ended questions addressing institutional organisation, professional background, and support activities related to research data management. Data cleaning was performed on the 77 valid entries collected (out of 88 responses) before conducting the descriptive analyses. Results indicate that most institutions employ staff performing data management support tasks which map to the definition of data stewardship, though these professionals are rarely formally recognised with that title. Hence, the variety of job titles observed reflects the absence of a standardised professional profile. Among the data management support tasks investigated, the most common include FAIR data management support, data management plan writing, researcher training, and policy consultancy. These findings provide the basis to establish the Italian Data Steward Community in order to overcome the fragmentation through peer-to-peer knowledge sharing and support, and to advance the definition of a professionalisation pathway for data stewards in Italy. The findings of this survey can serve as a case study to help data stewards, research data professionals, and policymakers in other countries to map and understand their local landscape.

## Plain Language summary

Good research depends on data that are well organised, well documented, and easy to share. Managing research data properly helps make research more transparent, trustworthy, and reusable, in line with Open Science principles. To support researchers with data management tasks, new professional roles are emerging. One of these roles is often called *data steward*, although this job title is not used consistently.

This study explores how data management support is organised in Italian universities and research institutions. Between April and December 2023, the Italian Computing and Data Infrastructure, supported by the Horizon Europe Skills4EOSC project, carried out a national survey to understand who provides data management support, what they do, and how they are recognised within their institutions.

The survey included 13 multiple-choice questions and was completed by 88 respondents, of whom 77 were suitable for analysis. The results show that many institutions already have staff members who support researchers with tasks such as writing data management plans, applying FAIR data principles, training researchers, and advising on data policies. However, these professionals often have different job titles and are rarely formally recognised as data stewards.

The study highlights a fragmented landscape and a lack of a clear professional profile in Italy. Based on these findings, the authors propose the creation of an Italian Data Steward Community to encourage knowledge sharing, reduce fragmentation, and support the development of a clear professional pathway. The results may also help other countries better understand and map their own data stewardship landscape.

## Introduction

Data stewards (DS) are a cornerstone of the research data ecosystem. Born at the intersection of data management, research support, and policy development, this profession has developed in response to the growing complexity of managing, sharing, and preserving digital research outputs. As research institutions have embraced the principles of Open Science and FAIR data ^1^ (Findable, Accessible, Interoperable, and Reusable data), the need for dedicated professionals who can bridge the gap between researchers, IT specialists, and policy-makers has become evident. DS are key actors in ensuring that research data are not only technically well-managed, but also ethically, legally, and contextually sound, and that research-performing institutions have in place policies and workflows that enable FAIR research data management (RDM), turning data into a true institutional and societal asset.

### International efforts to professionalise research data stewardship

Several efforts aim to align DS profiles and data stewardship service models at both the European and international levels. Within the Research Data Alliance (RDA), the Professionalising Data Stewardship Interest Group ^1^ (endorsed in 2020) has completed global surveys on data stewardship service models ( Ayres *et al.*, 2022) and DS career tracks ( Newbold *et al.*, 2024). Among those, a survey run in 2021 on DS service models found that most respondents considered their service provision still in the early stages of maturity ( Ayres *et al.*, 2022). Another one on DS career tracks, completed in 2022, highlighted that professionals who self-identify as DSs hold many different job titles and job roles – only 33% of the European respondents actually had the job title “data steward” – and underscored the lack of clearly defined career paths for DS ( Newbold *et al.*, 2024). Such surveys are not merely exploratory exercises; they play a crucial role in planning and developing practical implementations. For instance, the results of the RDA surveys were considered and incorporated by the European Open Science Cloud (EOSC) Task Force for Data Stewardship Curricula and Career Paths ( Kalová *et al.*, 2024). Similarly, the Horizon Europe Skills4EOSC project ^2^ built on the DS profiles, skills and competences defined in various national contexts to deliver Minimum Viable Skills profiles for “coordinating data steward” and “embedded data steward” that are intended to have European-level applicability ( Hansen *et al.*, 2025). All those efforts aim at the continued professionalisation of DSs and the maturing of data stewardship services in the research context.

### The landscape of research data stewardship in European countries

DS as professionals have emerged only in the last 10 or so years, at least in research contexts ( Wildgaard *et al.*, 2020). Following the publication of the FAIR data principles in 2016 ( Wilkinson *et al.*, 2016), the *Turning FAIR into reality* report (2018) by the European Commission’s Expert Group on FAIR Data recognised data stewardship as a key service for enabling FAIR data and proposed that new job profiles in data science and data stewardship are needed to support researchers throughout the research data lifecycle ( European Commission. Directorate General for Research and Innovation, 2018). Around the same time, universities in the Netherlands introduced their first dedicated data stewardship programmes, including the 3-year Data Stewardship Project in TU Delft that started in 2018 and introduced faculty-level DSs at the university ( Turkyilmaz-van der Velden, 2018). The years following the *Turning FAIR into reality report* also saw the launch of national-level projects in several European countries (e.g. the Netherlands, Denmark, Austria, and Germany) that surveyed local data stewardship landscapes, defined DS profiles and service models, identified skills and competences, and outlined training programmes ( Gruber, 2021; Jetten *et al.*, 2021; Seidlmayer *et al.*, 2023; Wildgaard *et al.*, 2020).

The DS profiles defined in these first projects highlight the multiplicity of functions that DS roles encompass. The Dutch and the Danish studies identified three key functional areas for data stewardship – policy, research, and infrastructure – and defined data stewardship roles in these areas ( Schmitz, 2020). The German study identified five DS profiles: (1) generalist, (2) advisory RDM, (3) discipline-specific, (4) coordinating, and (5) infrastructure-focused DS ( Seidlmayer *et al.*, 2023). There are overlaps between the Dutch, Danish, and German DS roles. However, the three studies also highlight that the roles and functions of a DS depend on the professional environment in which they operate. The Austrian study, in fact, directly links DS profiles to the professional environment by introducing professional profiles and related skills and competences through the definition of three data stewardship service models (contact point per faculty, central DS office, cross-organisation DS network) ( Gruber, 2021).

### Data stewardship in Italy

In Italy, similarly to other European countries, the role of the DS has not yet been formalised. Following the increasing importance of professional RDM activities to support the research lifecycle, several Italian universities and research institutions have progressively introduced similar institutional roles, training opportunities, and governance models for research data support. In 2019, a group of research and e-infrastructures in Italy signed the “Italian Computing and Data Infrastructure” (ICDI) ^3^ *memorandum of understanding*. ICDI is a forum created to promote synergies at the national level, and optimise the Italian participation in European and global challenges in the field of Open Science and FAIR data. By including several Italian research institutions and universities, ICDI progressively became the forum where Open Science and RDM experts could exchange ideas, start collaborations, and propose solutions to policy-makers with a bottom-up approach. The monthly online “Open Science Cafè” webinars, on major Open Science topics and updates, exemplifies ICDI's efforts in community engagement and building in the Italian landscape.

One of the leading organisations in ICDI is the Consortium GARR, which was also the coordinator of the aforementioned Skills4EOSC project. Among its activities, the project included the activation and support of professional networks of DSs to strengthen lifelong learning through peer exchange. Thanks to this favourable collaborative context created between ICDI and Consortium GARR within Skills4EOSC, the idea emerged to launch a public survey to map the state of data stewardship in Italian universities and research-performing institutions. A further objective of the survey was to assess the respondents’ willingness to join a national community of practice on RDM. This article presents the outcome of the survey and summarises the foundation of the Italian DS Community, a result of this first attempt to map data stewardship in Italy.

## Materials and methods

### Survey development and distribution

Under the umbrella of the Italian Computing and Data Infrastructure (ICDI), Consortium GARR, as the coordinator of the Skills4EOSC project, together with the University of Bologna (UNIBO) and the Italian Institute of Technology (IIT), designed and distributed the “Survey on Data Stewards in Italy: a mapping of professionals supporting research data management”.

The survey was developed as a web-based set of questions using EUSurvey, an open-source platform by the European Commission. It was distributed in Italian to facilitate completion and improve response rates, but an English version was also prepared.

Dissemination followed a snowball sampling approach, beginning with targeted outreach, through two national mailing lists focused on Open Science (“ oa-italia@lists.icdi.it” and “ icdi@lists.garr.it”), and expanding the audience through word-of-mouth, institutional social media channels (e.g., Skills4EOSC linkedin page ^4^), and an article in GARR Magazine ( Colcelli *et al.*, 2023).

The survey was officially launched in April–May 2023 (from 13/04/2023 to 31/05/2023) and reopened in November–December 2023 (from 07/11/2023 to 01/12/2023).

### Survey and question structure

To structure the data collection and facilitate analysis, the survey was divided into three main sections:

•   1. Introduction: Provided a general definition of “data steward” to ensure a shared understanding among respondents.

•   2. Respondent Profile: Collected information about the respondent’s institution, role, and whether the response was personal or institutional.

•   3. Institutional Characterisation: Included 13 closed-ended questions to explore how the DS role is defined and how related services are structured in the respondent’s institution.

Only those respondents who reported DSs or equivalent roles in their institutions could access the third section of the survey; for all others, the survey ended after collecting profile information.

The survey consisted of three types of questions, distributed across the sections described above. Open-ended questions were included to capture information that could not be reliably categorised in advance. In addition, we incorporated multiple-choice questions where respondents were required to select one mutually exclusive option, as well as multiple-choice questions that allowed the selection of more than one response.

The survey was designed to collect only anonymous data from participants.

Both templates, in English and Italian, as well as the dataset, have been archived in Zenodo ( Caldoni *et al.*, 2025a).

### Data cleaning and visualisation

A total of 84 anonymous responses were collected and exported as a .csv file, on which data cleaning was performed.

OpenRefine (Open Refine, v3.8.7) was used to split multiple-choice responses, correct inconsistencies and cluster roles into three macro-categories: “researcher”, “technologist” and “technical-administrative staff”. These three macro-categories were chosen as they represent distinct and formalised functions in Italian universities and research institutions. Namely, “researchers” are directly responsible for producing scientific knowledge and conducting original research, while “technologists” provide advanced technical and specialist support to research. This second role typically involves managing laboratories, digital infrastructures, data platforms, scientific instruments, or complex technical workflows. Finally, “technical-administrative staff” ensures the day-to-day functioning of the institution by handling administrative, financial, and organisational tasks.

Manual data cleaning operations consisted of:

•   adding ROR identifiers when not provided by respondents,

•   removing responses not from Italy (used for functionality tests),

•   adding a column for institutional acronyms,

•   classifying institutions (universities vs. research entities),

•   eliminating duplicate answers.

Lastly, two responses referring to the respondents’ affiliated institutions were manually inspected and excluded because the authors could unequivocally verify that these records were incorrect.

After cleaning, 77 valid responses remained. The final dataset was reviewed by all authors to ensure consistency and accuracy.

Given the limited sample size, the analysis was restricted to descriptive statistics, performed using Microsoft Excel (Microsoft Corp., Redmond, WA, USA).

**Figure 1.**
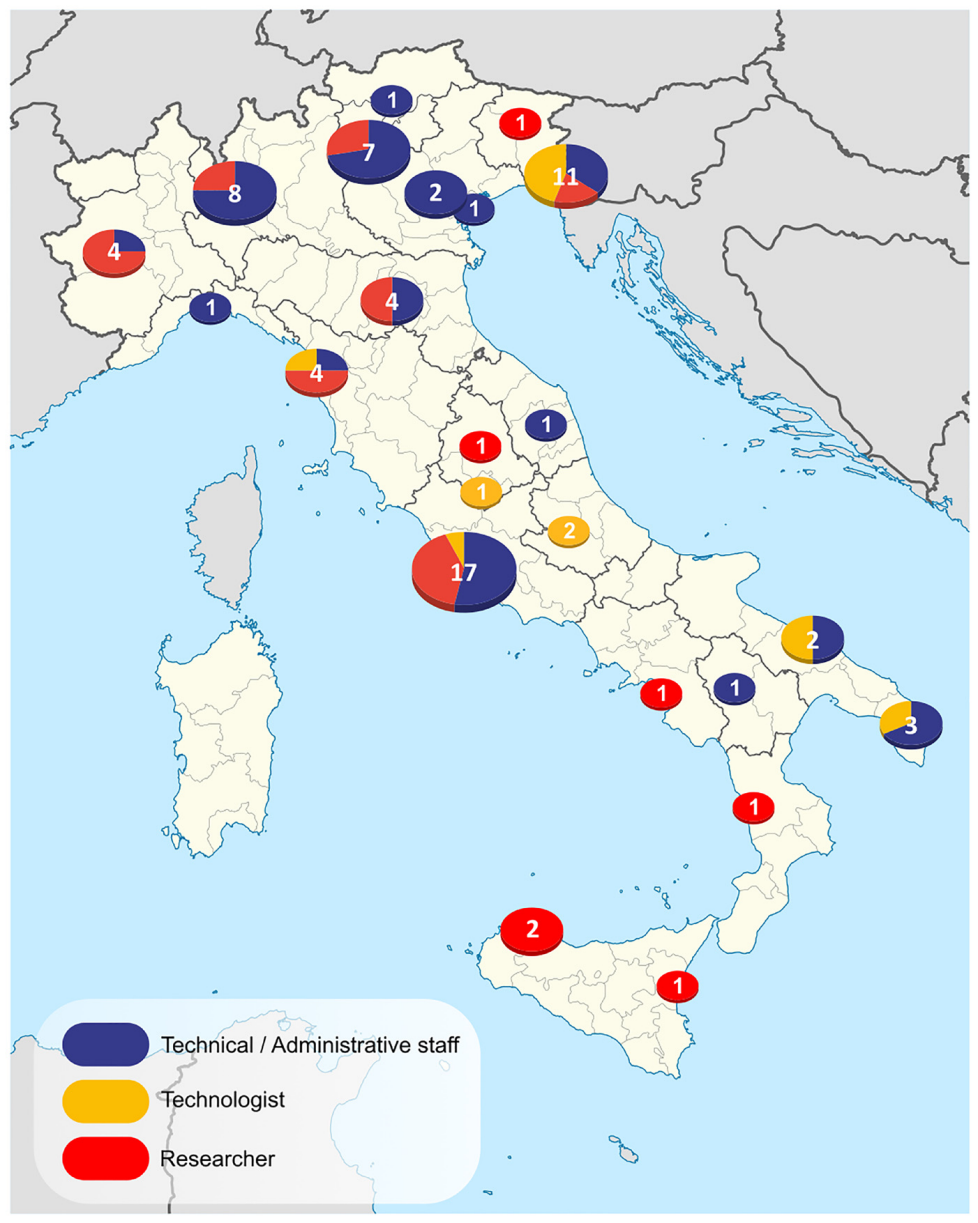
Map of institutional roles represented among respondents to the national survey.

In-house built R code was developed to produce a map of the geographical distribution of the respondents ( Figure 1). The resulting R-plot was replicated, for visualisation purposes, into a multi-level image using vector graphics editor Inkscape ^5^. To improve readability, city-centred pie-charts reflecting geographical provenance were subdivided into 4 categories, according to the number of respondents. Cities with a single respondents were chosen as the unit size. Pie charts for cities with 2–5 respondents were visualised as 1.5 times the size of the unit; for cities with 6–14 respondents, the pie chart size was scaled at 2 times the size of the unit. The only pie chart corresponding to a city with more than 14 respondents was resized to 2.5 times the size of the unit.

The results of the analysis are presented in graphs, maps and tables to visualise the diversity and geographical coverage of the survey.

## Analysis of survey results

### Institutional roles and personal interests

First, we asked respondents to indicate their institutional affiliation and specify their role within the institution. This allowed us to have a better picture of the professionals involved, which was useful in the qualitative analysis of the data. 33 respondents are affiliated with universities, while 44 are affiliated with public research institutions ( Figure 1).

Regarding the professional role of the respondents, we considered the three main types of employees within the Italian public research institutions: Technical/Administrative staff, Technologist, and Researcher. To highlight possible geographical differences within the respondents' population, we collated responses from different research institutions belonging to the same city. We mounted single-city pie charts on a map of Italian administrative subdivisions ^6^.

The majority of the respondents belong to the Technical/Administrative support staff (n=38), while another important portion of respondents identified themselves as a Researcher (n=27). Only a small number of Technologists (n=8) answered the survey, consistent with the uneven distribution of this professional framework across the national landscape (see the section “Data cleaning and visualisation” in the “Materials and Methods” chapter for the definition of these three different professional roles).

**Figure 2.**
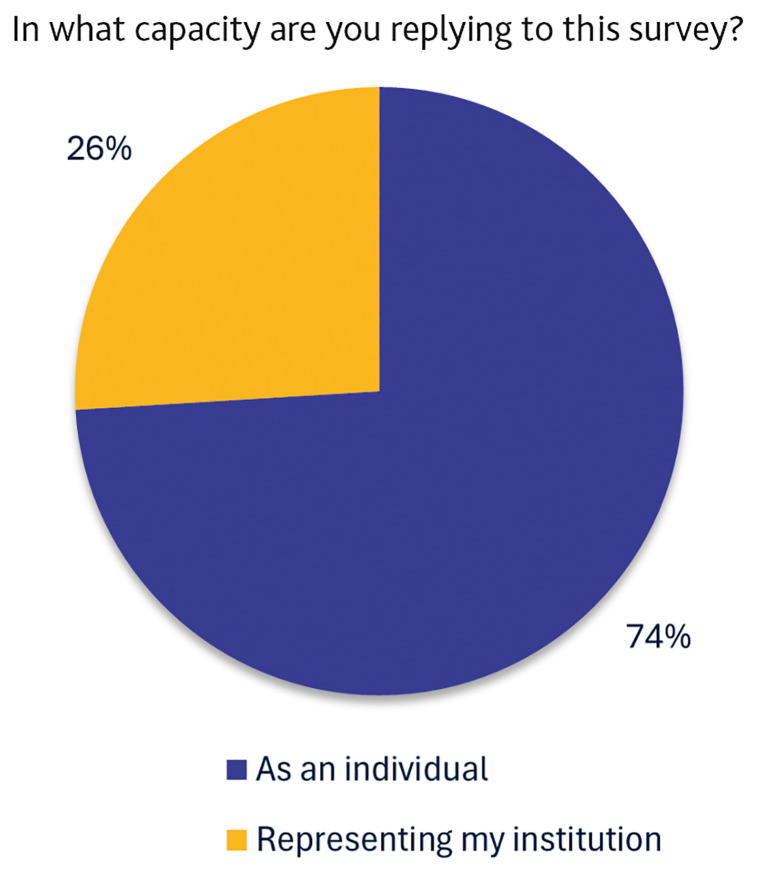
Distribution of respondents according to the capacity in which they completed the survey. (n=77 respondents.)

Then, we asked whether the respondents were participating in the survey in a personal capacity or whether they were officially representing their institution. Most of the respondents participated in a personal capacity (about three quarters), as presented in Figure 2.

### Formal recognition of Data Stewards

We started our analysis by examining the presence and the role of DSs or analogous figures within the institutions that participated in the survey. In this section, we focus our analysis solely on the responses provided on behalf of the institution (n=20).

**Figure 3.**
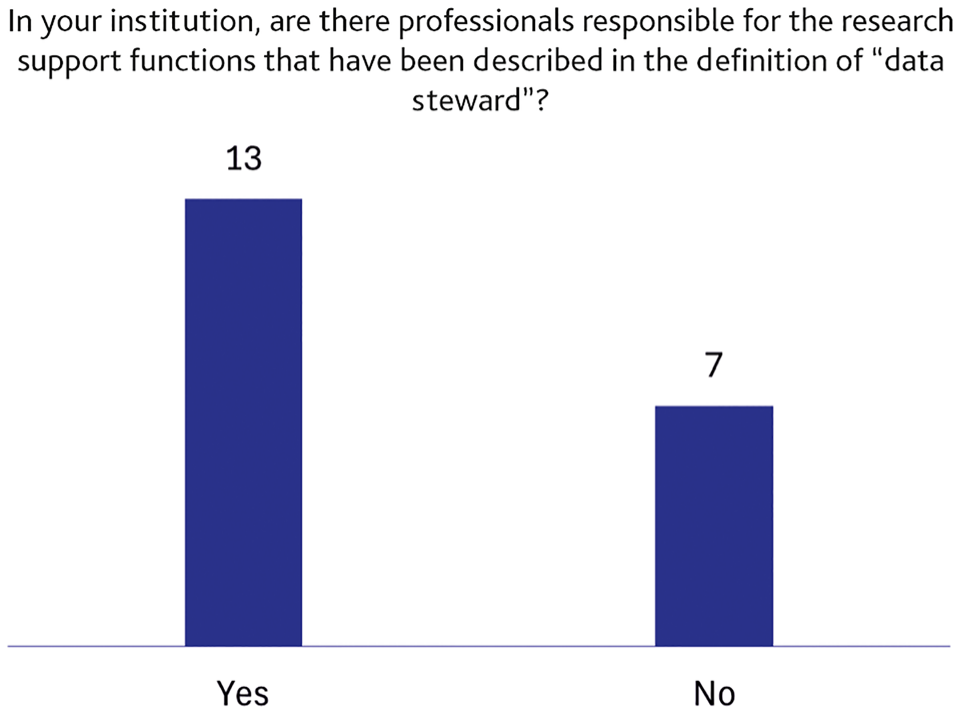
Presence of professional figures performing research support functions described in the definition of “data steward”. (n=20 respondents, answering on behalf of their institutions.)

About 65% (13 out of 20) of the respondents state that there are professionals who assume the role of DS ( Figure 3).

**Figure 4.**
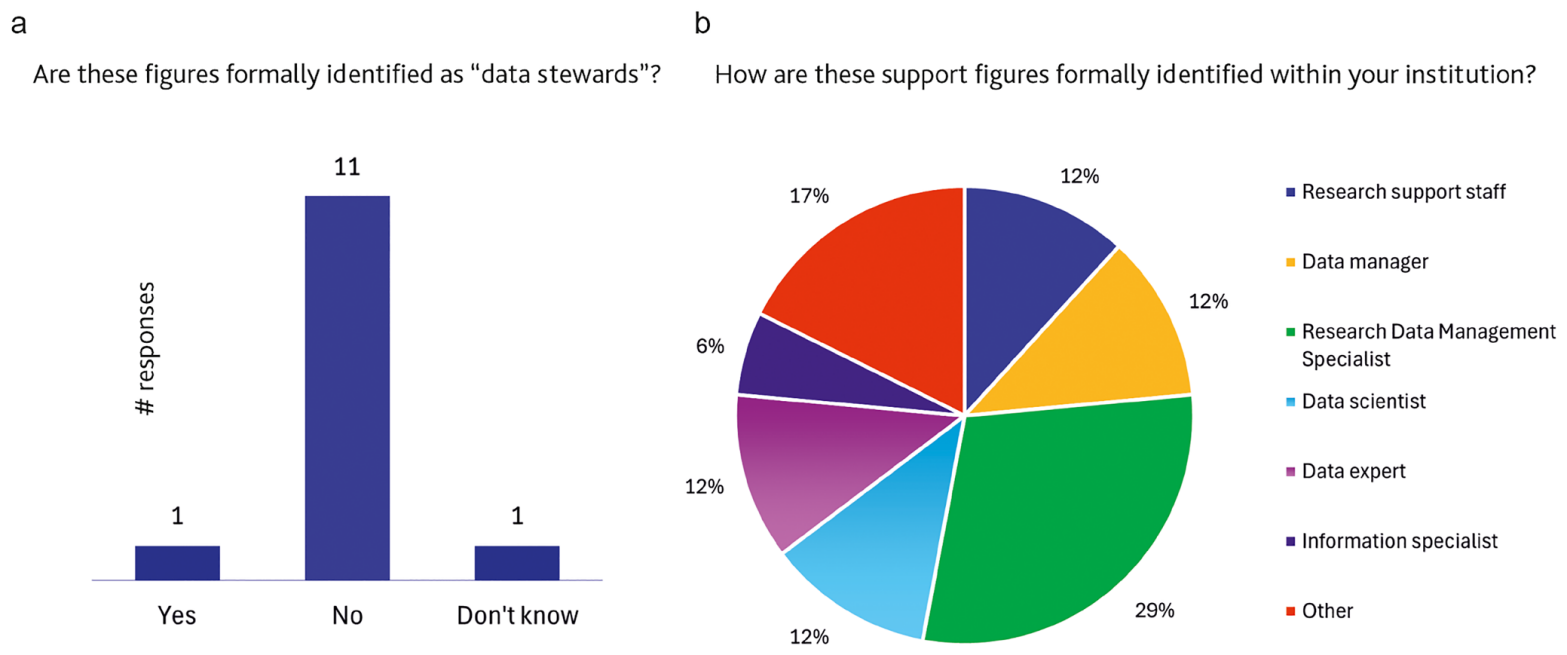
**a**. **Presence of professional figures supporting research management formally identified as data stewards.** (n=13 respondents, answering on behalf of their institutions and reporting that the institution has staff dedicated to the support functions described in the definition of a “data steward”.) **b**. **Breakdown of the formal designation of research support figures within institutions.** (n=11 respondents, answering on behalf of their institutions and reporting that the institution has staff dedicated to the support functions described in the definition of a “data steward” but not formally identified as such. Multiple (non-mutually exclusive) responses allowed.)

Interestingly, when participants were asked which type of position is held by individuals acting as DS, only one in 13 respondents reported that the role is formally recognised ( Figure 4a).

Figure 4b shows that the job titles used to identify professional figures working in data management support roles can vary considerably, even within the same institution. Respondents were allowed to select as many job titles as necessary to describe the reality of data management support in their institutions. Respondents were provided with seven possible, non-mutually exclusive answers, partially derived from the roles most represented in the international landscape ( Ayres *et al.*, 2022; Jetten *et al.*, 2021) and had the chance to add more options in a free text box if necessary.

**Figure 5.**
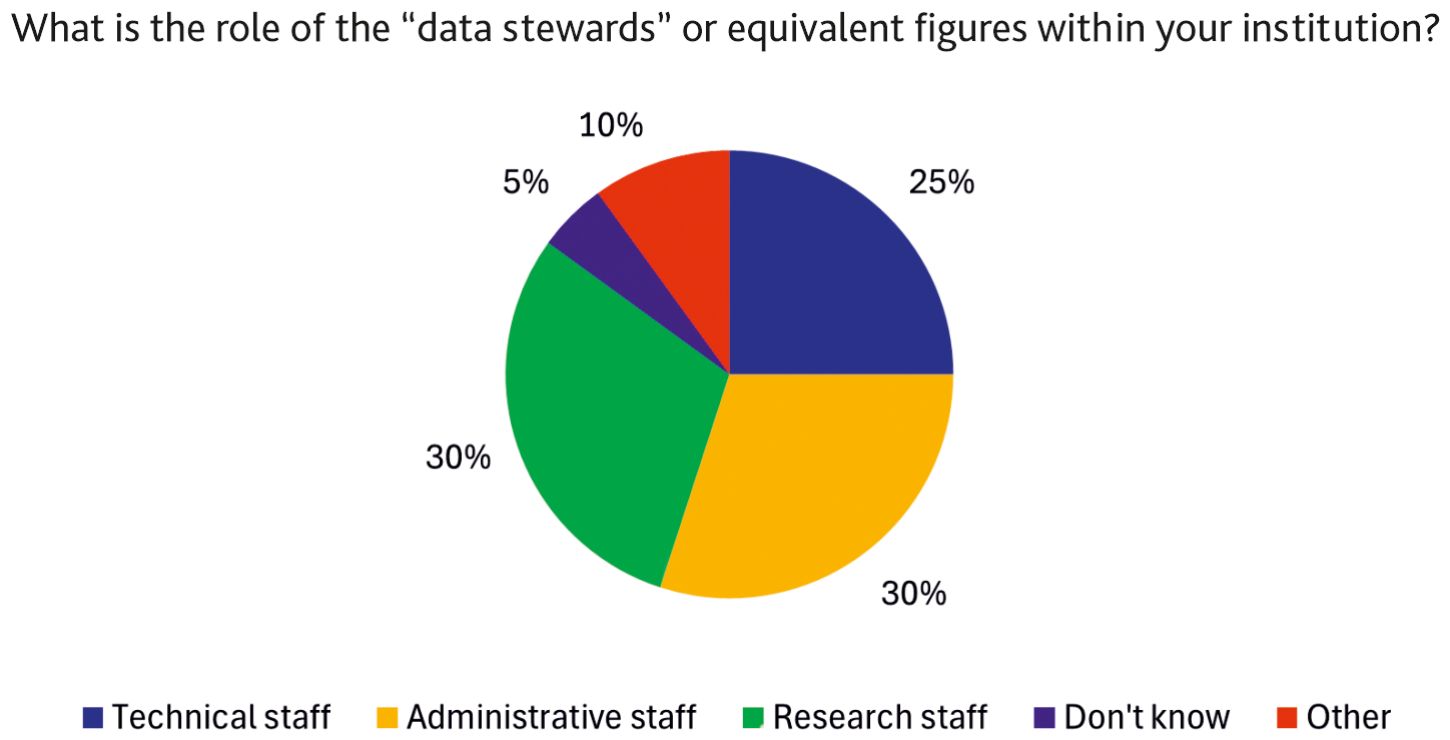
Breakdown of the roles of data stewards or analogous figures within institutions. (n=13 respondents, answering on behalf of their institutions and reporting that the institution has staff dedicated to the support functions described in the definition of a “data steward”. Multiple (non-mutually exclusive) responses allowed.)

Further evidence of the lack of a clear professional framework for DS is seen in the distribution of the roles for those performing data stewardship activities, including research roles (post-doc, research fellow, etc.), technical roles (e.g. IT support, data processing technician, etc.) and administrative roles (e.g. librarian, research support, project manager and others). As shown in Figure 5, no significant differences were observed across these categories.

### Background, training and activities

Considering the lack of any officially recognised national training pathway leading to the title of “data steward” in Italy, we asked about the educational background of DSs or equivalent figures.

**Figure 6.**
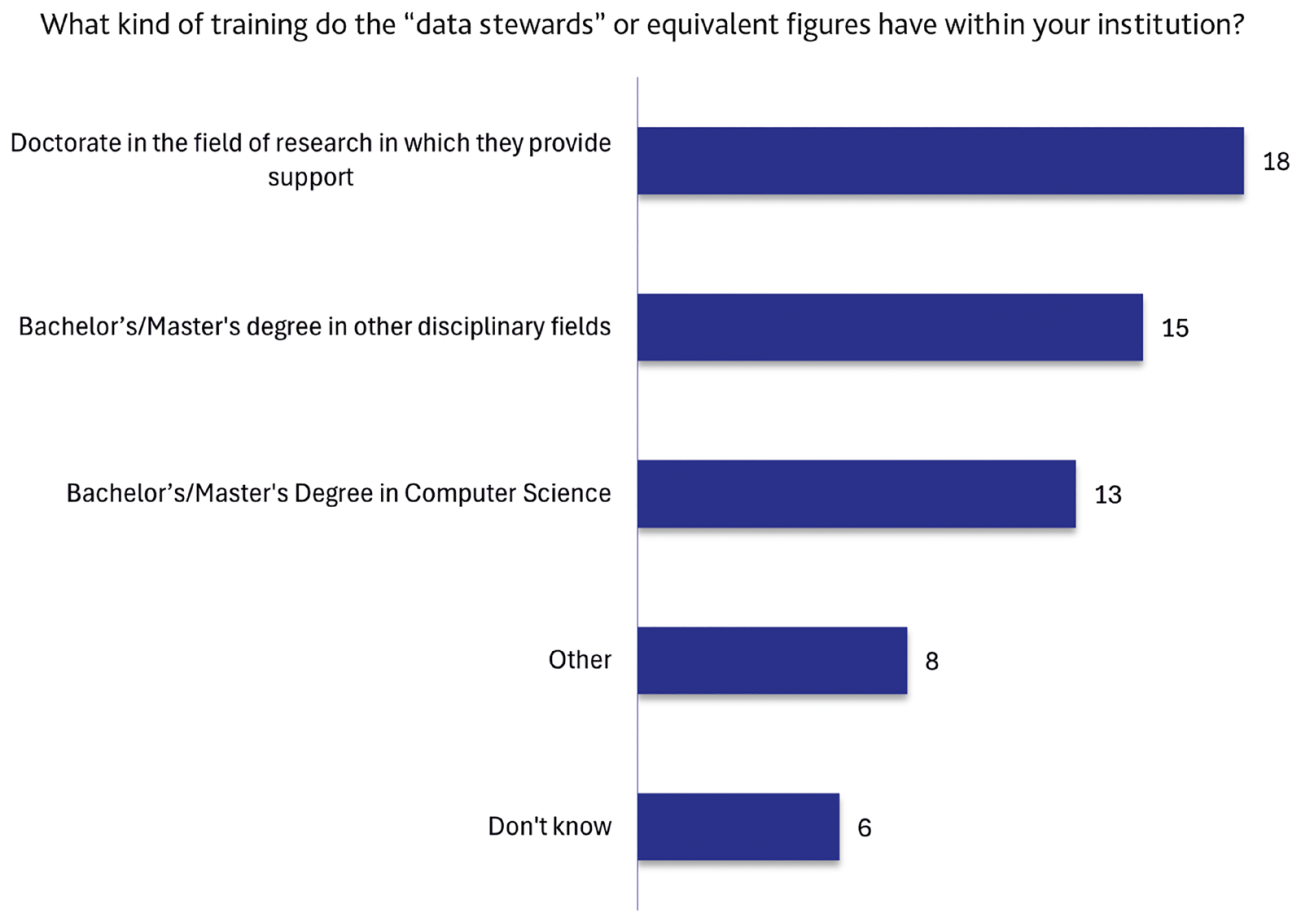
Breakdown of the types of training possessed by data stewards or equivalent figures before their recruitment within institutions. (n=38 respondents, answering either on behalf of their institution or personally, reporting that the institution has staff dedicated to the support functions described in the definition of a “data steward”. Multiple (non–mutually exclusive) responses allowed.)

Figure 6 shows the prevalence of specific disciplinary backgrounds. Indeed, the largest group (18 responses) reported a master’s degree or PhD in a relevant disciplinary field (e.g., biology, physics, social sciences). The second largest group (15 responses) indicated degrees in related or complementary fields (e.g., library science, archiving, information science, statistics). A total of 13 respondents reported degrees in Computing or Computer Science. Finally, 8 responses selected “Other” and 6 selected “I don’t know”.

### Data Stewardship activities

After detailing the DSs’ background, we investigated their activities, focusing mainly on support and data management ( Ayres *et al.*, 2022; Basalti *et al.*, 2024; Jetten *et al.*, 2021). Namely, we asked what kind of support the DS or equivalent figure provides within their institution, providing seven possible answers and the option to indicate more functions. The list of proposed answers was:

•   “support for research data management and the application of FAIR principles, identification of disciplinary repositories, storage and backup solutions, metadata, etc.;”

•   “support for writing the data management plan; assessment of possible legal issues (e.g., privacy, IP and licences, etc.);”

•   “drafting of institutional policies for research data management;”

•   “advice on national, international and funding body policies and mandates for data management and compliance with any obligations;”

•   “training of researchers on Open Science, FAIR data and research data management issues;”

•   “support for the use of data management tools made available by their institution (e.g. institutional repository, institutional cloud, etc.);”

•   “support for the management and maintenance of the institutional repository (e.g. care and validation of deposited datasets).”

Respondents could select more than one answer in order to capture the extent to which DS activities are characterized by specialization or encompass multiple functions.

**Figure 7.**
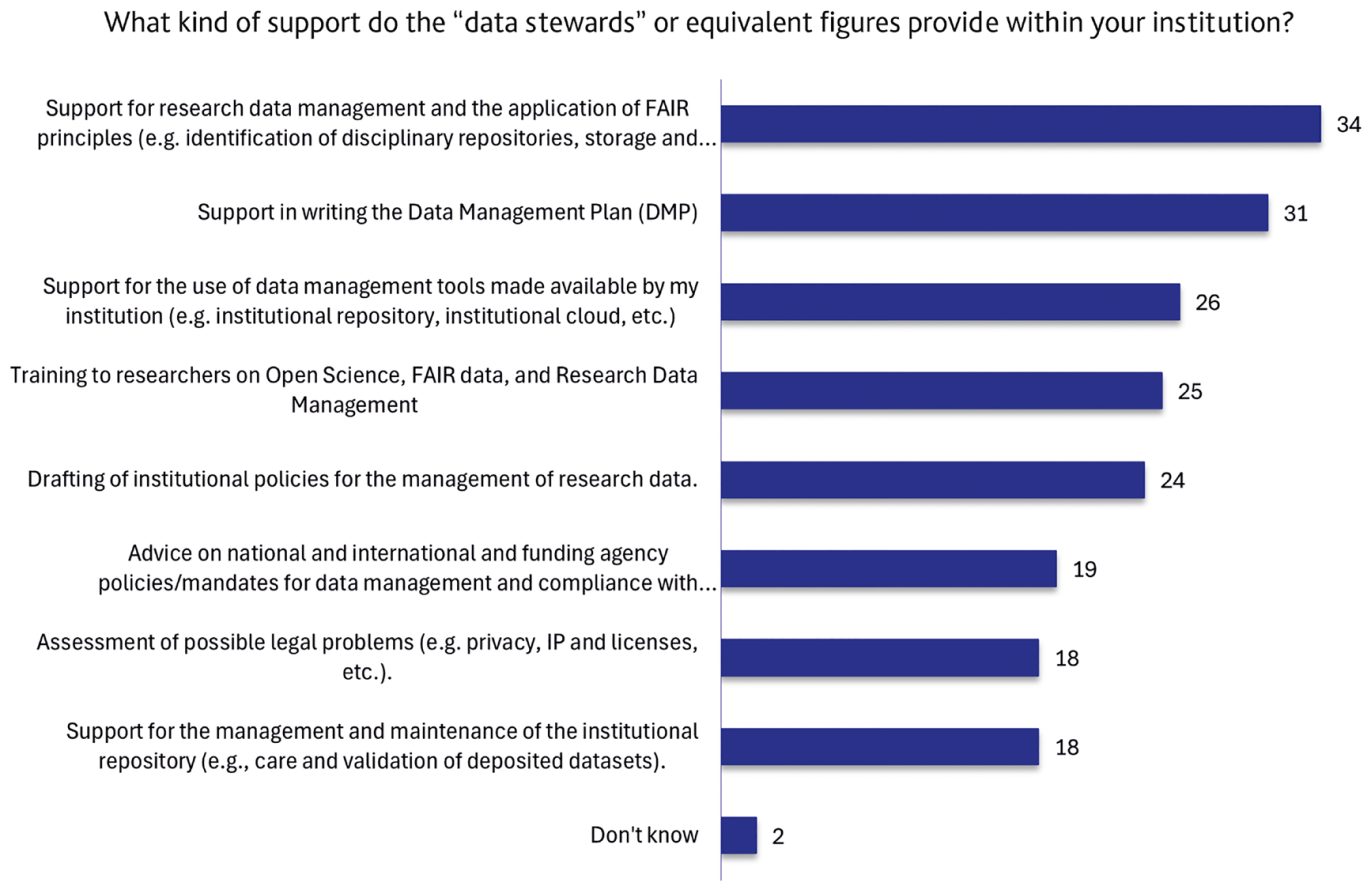
Breakdown of the support functions carried out by data stewards or analogous figures within institutions. (n=38 respondents, answering either on behalf of their institution or personally, reporting that the institution has staff dedicated to the support functions described in the definition of a “data steward”. Multiple (non–mutually exclusive) responses allowed.)

The results ( Figure 7) show that DSs or equivalent figures are prevalently involved in RDM support, particularly in applying FAIR principles (34) and assisting with DMPs (31 responses). A second cluster of activities is represented by drafting institutional policies (24 responses), training on Open Science and RDM (25), and support for institutional data management tools (26). More specialised activities like providing advice on national, international and funding body policies and mandates for data management and compliance with any obligations (19), as well as legal assessment (18) and repository maintenance (18) show a moderate engagement.

As shown in Figure 7, most of the respondents (95%) mentioned at least 1 support task out of the 8 we proposed in the survey. Most respondents indicated a combination of tasks: only 6 respondents (16%) mentioned 1-3 related tasks. The remaining part of the respondents (30 out of 38, more than 75% of the cohort) mentioned at least 4 tasks. This multi-task related partition shows a substantial equality between the number of tasks: the number of people mentioning 4 or more tasks (up to 8) is, for all the 5 categories (4,5,6,7,8 tasks), always about 15% (respectively, 6,5,5,7,7 people). By focusing on the 11 (about one third of the cohort) surveys reporting 4-5 tasks, there is a solid pattern (10 out of 11) mentioning both RDM and DMP. This often (7 people) occurs together with training duties. A substantial proportion (50%, 19 respondents) of the entire cohort enumerated 6 or more different tasks. They covered most of the proposed tasks, with slightly fewer mentions (13 out of 19) in "Advice on local and international policies" and "Institutional repository" tasks.

After mapping the type of support provided by DSs or analogous figures, we decided to investigate the target groups of their services. In particular, we asked whether they were limited to researchers with data management requirements derived from projects or if they were extended to all researchers. A striking majority of 29 respondents (76%) indicated that services are primarily aimed at all research staff, regardless of project affiliation or funding source, while in just 6 cases (16%) a restriction was reported.

### Analysis of data stewardship services and professional classification areas

After collecting information on the formal recognition, background, and activities of DSs and similar roles, we proceeded to investigate how this role is characterised across institutions. We therefore evaluated both the number of such professional figures employed in each organisation and the professional level at which they are positioned, based on the responses indicating their presence within the institution.

**Figure 8.**
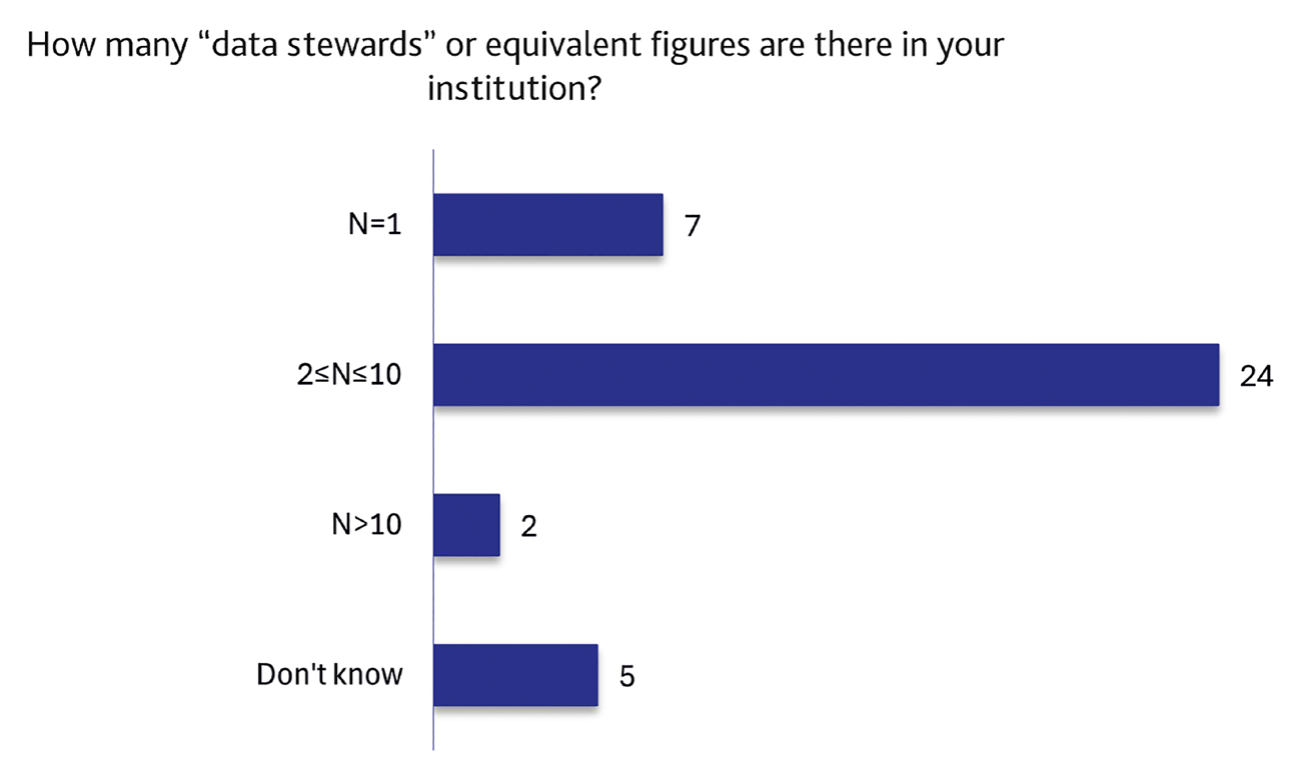
Count of “data stewards” or equivalent figures within institutions. (n=38 respondents, answering either on behalf of their institution or personally, reporting that the institution has staff dedicated to the support functions described in the definition of a “data steward”.)

As shown in Figure 8, most respondents reported that their institution had two or more professionals performing roles equivalent to DSs. Only a minority of institutions reported the presence of a single individual in this role.

**Figure 9.**
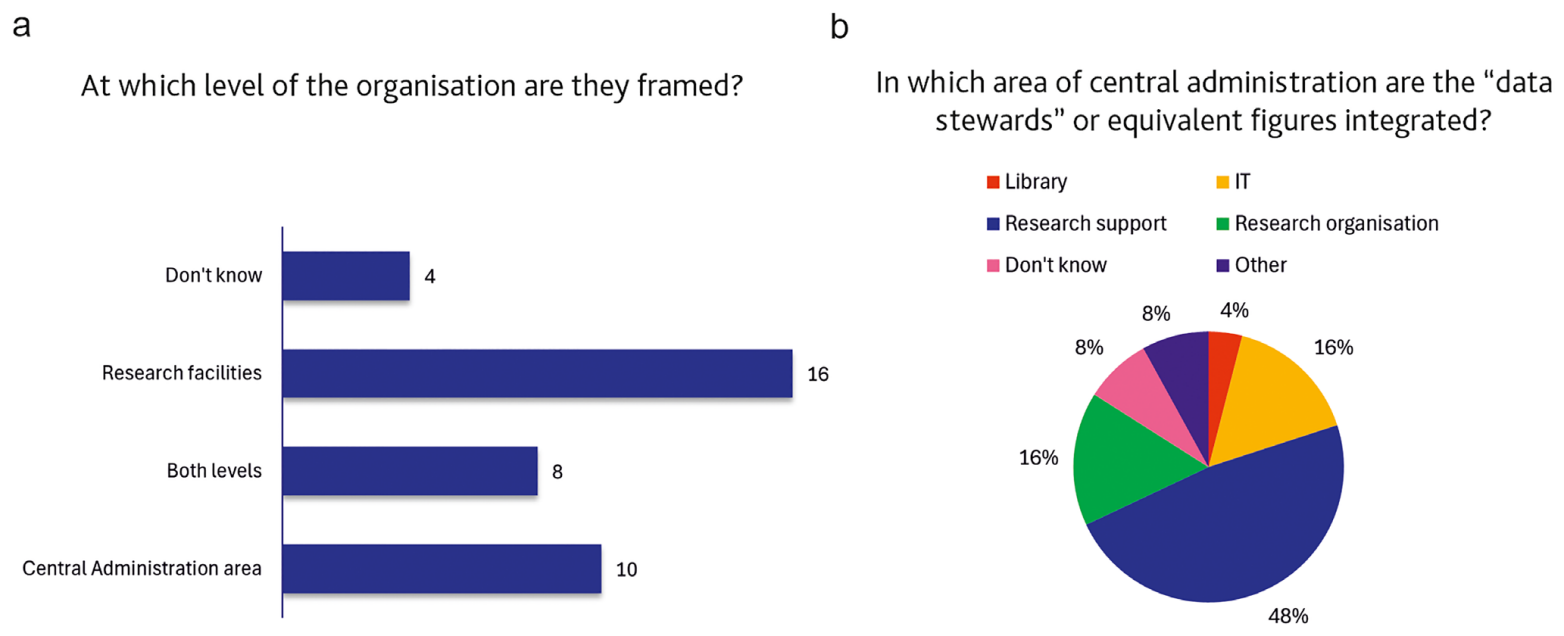
**a**. **Level of the organization where “data stewards” or equivalent figures are framed within institutions.** (n=38 respondents, answering either on behalf of their institution or personally, reporting that the institution has staff dedicated to the support functions described in the definition of a “data steward”.) **b**. **Breakdown of the central administration areas where "data stewards" or equivalent figures are framed within institutions.** (n=18 respondents, answering either on behalf of their institution or personally, reporting that the institution has staff dedicated to the support functions described in the definition of a “data steward” and employed, either exclusively or partially, in a central administration area. Multiple (non–mutually exclusive) responses allowed).

Figure 9a shows that in approximately 40% of cases (16 out of 38) DS are embedded within research facilities, while a substantial proportion of responses frame these professional figures within central administrative areas. Interestingly, about 25% of respondents reported a hybrid situation, with DS framed at both levels. Figure 9b shows that the majority of DSs positioned within central administration are associated with:

•   Research Support Offices or Research Management and Administration (RMA) units;

•   IT and Infrastructure Services, especially where data repositories or digital infrastructures are managed;

•   Libraries or Documentation Services, in a smaller number of cases.

Since this question allowed for multiple, non-mutually exclusive answers, we can report that a few responses mentioned hybrid or cross-departmental structures, where DSs collaborate between research support, IT, and administrative divisions: this may reflect the interdisciplinary and cross-functional nature of the role, even within centralised structures.

## Discussion

The findings of this survey provide an overview of the emerging professional landscape of data stewardship in Italian universities and public research institutions.

We acknowledge the limitations of this analysis, which is based on data collected in 2023 and may not fully reflect the current situation. Although the survey allowed us to gather responses from across the country ( Figure 1), we recognise that they come only from a limited number of Italian research-performing institutions ^7^. Despite these shortcomings, the results highlight that the role is increasingly recognised as essential for supporting research, yet still characterised by fragmented professional identities, heterogeneous role definitions, and limited institutional recognition. In the future, we plan to perform another survey including as many universities and research institutions as possible. It would also be interesting to explore the private sector, which was not targeted in this initial survey, as it was disseminated through mailing lists which primarily reach academic and research personnel. To establish a common basis for the subsequent questions, we defined DS in the introductory section of the survey ( Demchenko & Stoy, 2021; Jetten *et al.*, 2021; Wildgaard *et al.*, 2020). This was also intended to support respondents who might not be familiar with or fully confident with this professional figure. Interestingly, approximately 65% of the respondents reported the presence of professionals performing DS-related activities ( Figure 3). This finding confirms the importance of professionalising this role, as it shows that DS-like tasks are already embedded in the everyday functioning of many Italian universities and public research institutions. However, this is also coupled with a lack of institutional recognition. As shown in Figure 4a, only one out of thirteen respondents indicated that positions performing DS-related tasks are formally labelled as “data steward”. This finding is consistent with the results of previous surveys ( Newbold *et al.*, 2024), which similarly reported limited formal recognition of the role. The variety of job titles associated with DS-like responsibilities, even within the same institution ( Figure 4b), highlights significant fragmentation and aligns with recent literature emphasising the lack of role standardisation at national and European levels ( Ayres *et al.*, 2022; Hansen *et al.*, 2025). Similarly, the distribution across research, technical and administrative roles ( Figure 5) suggests that although the activities associated with data stewardship are increasingly needed, they are often assigned to existing roles without providing adequate formalisation, visibility, or clear career pathways, which can also result in differences in remuneration, as DS performing similar tasks in different institutions may fall under positions with varying salary scales.

Conversely, it is notable that about 35% of respondents ( Figure 3) stated that no specific personnel in their institution is dedicated to any of the RDM activities indicated, suggesting an uneven adoption of DS practices across the national landscape. At this stage, we are not able to determine the exact reasons for this, but we can hypothesise that it may reflect insufficient investment in RDM support services or that DS-like activities are performed informally or distributed across multiple roles, making them difficult for respondents to identify.

Consistent with European trends showing that institutional maturity in RDM correlates with the creation of dedicated professional roles ( Gruber, 2021; Jetten *et al.*, 2021; Seidlmayer *et al.*, 2023; Wildgaard *et al.*, 2020), only a minority of respondents participated on behalf of their institutions ( Figure 2). It is reasonable to infer that institutions demonstrating a higher level of awareness and engagement with RDM topics are those that have designated a representative to respond on behalf of the institution. These institutions are also more likely to be in the process of developing or implementing structured support services for RDM.

On the other hand, the large proportion of respondents who answered in a personal capacity may reflect limited institutional awareness/policies related to RDM needs or a lack of internal communication about existing practices.

These data further support the idea that the concept, functions and activities of a DS often depend heavily on the institution in which they work ( Jetten *et al.*, 2021; Seidlmayer *et al.*, 2023; Wildgaard *et al.*, 2020). While in some organisations DS-like professionals have gradually been integrated, even if their roles are not formally recognised or consistently defined, in other institutions it appears that no one is explicitly responsible for RDM-related tasks. This variability underlines the absence of a coordinated national framework and highlights how institutional context shapes the way data stewardship is interpreted and implemented within the Italian research system.

Part of the problem might be related to the difficulties in recognising who qualifies as a DS, as there is currently no universally accepted definition.

According to the responses that we gathered here, a DS is a professionals with (at least one or more) cross-cutting skills in RDM (disciplinary, IT and technical, legal) and who very often acts as a bridge between researchers (i.e. producers and users of research data), infrastructures and research organisations ( Figure 6 & Figure 7). This scenario is in line with previous national-level surveys conducted in Europe ( Ayres *et al.*, 2022; Gruber, 2021; Jetten *et al.*, 2021; Seidlmayer *et al.*, 2023; Wildgaard *et al.*, 2020).

The analysis of educational backgrounds ( Figure 6) provides further evidence of a fragmented professional identity. In the majority of cases, DSs hold advanced degrees in disciplinary fields related to their data stewardship activities. This observation supports a DS model in which the direct research experience is extremely relevant to shape the dialogue with the main stakeholders of data stewardship services, the researchers, and aligns with previously reported international experiences ( Gruber, 2021; Jetten *et al.*, 2021; Seidlmayer *et al.*, 2023; Wildgaard *et al.*, 2020). Additionally, the notable representation of respondents with training in computing or computer science indicates a growing emphasis on the technical dimension of the role. Nonetheless, the number of responses classified as “Other” or “I don’t know” reflects a lack of consensus regarding the educational pathways necessary for acquiring DS competences and confirms the absence of nationally recognised training standards.

Despite the fragmentation in roles and backgrounds that characterise the figure of a DS, we observed a broad consensus in terms of support activities that they provide within their institution. The survey offered a selection of eight activities that a DS can potentially perform ( Figure 7), and support for RDM and data management plan drafting were the two most frequently selected tasks. Interestingly, these core tasks are often carried out alongside two or more additional activities, usually related to the support in implementing the institutional policies and tools in everyday research work, often complemented by training and capacity-building efforts. These results highlight that the role of the DS requires multiple skills, at the intersection between those specific to the research domain and those typical of administrative functions. This peculiarity differentiates this profession from the fields of project management and IT management and adds another layer of complexity to the role. Notably, only three out of 38 respondents added new activities beyond those proposed, which could still be attributed to them, suggesting that the list provided in the survey was exhaustive and reflects the key functional areas perceived as central to data stewardship in the Italian research context.

Building on the observation that DS-like responsibilities are already embedded in day-to-day activities of many Italian universities and public research institutions, we were interested in reporting the number of DS-like professionals within each organisation ( Figure 8) and their organisational placement ( Figure 9a and 9b).

Most respondents reported that their institution hosts between two and ten DS-like professionals. While this category is very broad and does not allow for an accurate estimation of the number of DS in Italian research institutions, it tells us that there is often more than one figure associated with DS-like activities. The next most common response indicated the presence of only one DS-like professional per institution, suggesting that in many cases, support for RDM remains limited or concentrated in a single individual. It is also noteworthy that five respondents declared that they did not know how many DS-like professionals were present in their organisation. This uncertainty further highlights the limited visibility of these roles and suggests that institutional communication regarding the existence, distribution and responsibilities of DS-like staff is probably still insufficient in many contexts.

The survey findings reveal that DS-like professionals are primarily based in research facilities, yet the overall numbers are similar to those of DS framed in central administration. This dual distribution is coherent with the coexistence of two different organisational models, the “coordinator” and the “embedded” DS, which were recently coded in the Minimum Viable Skillset produced by the Skills4EOSC project ( Hansen *et al.*, 2025). In particular, the project defines the “Coordinator” DS as the one providing support across an organisation’s research domains and units, while “Embedded” DS operates close to a research team and to its domain-specific practices. Interestingly, around 20% of respondents (8 out of 38) reported that DS are framed at both levels in their institutions, proving that some Italian institutions are still experimenting with hybrid organisational frameworks that can benefit the general academic community while also providing specialised support.

Overall, this study offers an overview of the fragmented and still evolving landscape of data stewardship in Italy. The findings may support institutional decision-makers not only in defining and structuring data stewardship services within an increasing number of universities and research institutions, but also in advancing the professionalisation of the role and the development of dedicated career paths. Beyond its immediate results, the survey represents a valuable output in itself, as its methodology can be readily adopted in other national contexts seeking to undertake a similar landscape analysis.

Importantly, the survey also served as the initial step towards the establishment of the Italian Data Stewards Community (Comunità Italiana Data Steward, CIDS) in 2023. The Community has since articulated three overarching objectives, formalised in its 2025 manifesto ( Caldoni *et al.*, 2025a): (i) to create opportunities for sharing and developing new skills; (ii) to support the adoption of best practices in RDM; and (iii) to increase awareness of the DS’s role within the research ecosystem. As a bottom-up initiative, the Community now includes more than 150 members and promotes networking and knowledge exchange through regular meetings, publications, and collaborative activities. The survey thus played a foundational role in fostering the Community’s self-recognition and professional consolidation within the Italian context.

## Ethics and consent

Ethical approval and consent were not required for this submission.

## Data Availability

Zenodo: Data and code for Mapping Data Stewardship in Italy: Findings from the First National Survey (1.0). https://doi.org/10.5281/zenodo.17907703 ( Caldoni *et al.*, 2025b) The project contains the following underlying data: Data_Figure1.csv (Portion of the whole dataset, integrated with geographical information related to the institutions of the respondents, ready to be used by “Make_Figure1.R script) Data_Survey.csv (File containing the whole dataset acquired through the survey) DS_Survey_Template_EN.pdf (English version of the text of the survey presented to the participants) DS_Survey_Template_IT.pdf (File containing the text of the survey presented to the participants) Make_Figure1.R (R code to produce Figure 1 on “Mapping Data Stewardship in Italy: Findings from the First National Survey” manuscript) Readme.pdf (Readme file describing the content of the dataset)

## Footnotes

^1^ RDA Professionalising Data Stewardship Interest Group, https://www.rd-alliance.org/groups/professionalising-data-stewardship-ig/activity/, URL checked on 2025/11/28

^2^ Skills for the European Open Science Commons: creating a training ecosystem for Open and FAIR science, https://www.skills4eosc.eu/, URL checked on 2025/12/04

^3^ ICDI, Italian Computing Data Infrastructure, https://www.icdi.it/en/, URL checked on 2025/12/04

^4^ https://www.linkedin.com/company/skills4eosc/, URL checked on 2025/12/11

^5^ Inkscape 1.3 (0e150ed6c4, 2023-07-21), https://inkscape.org, URL checked on 2025-24-10

^6^ https://commons.wikimedia.org/wiki/File:Italy_provincial_location_map_2016.svg, URL checked on 2025/10/24

^7^ https://www.mur.gov.it/it/aree-tematiche/universita/le-universita, URL checked on 2025/12/02 https://www.mur.gov.it/it/aree-tematiche/ricerca/il-sistema-della-ricerca/enti-di-ricerca-pubblici URL checked on 2025/12/02
