# Data stewardship in Italy: Findings from the first national survey

**DOI:** 10.12688/openreseurope.22183.2

**Published:** 2026-07-31

**Authors:** Giulia Caldoni, Vittorio Iacovella, Emma Lazzeri, Liise Lehtsalu, Francesca Marchegiani, Mauro Paschetta, Valentina Pasquale, Giulia Pedonese, Andrea Tarallo

**Affiliations:** 1 Research Division (ARIC), Alma Mater Studiorum - Università di Bologna, Bologna, Emilia-Romagna, 40126, Italy; 2 Universita degli Studi di Trento Centro Interdipartimentale Mente/Cervello, Rovereto, Trentino-Alto Adige/South Tyrol, 38068, Italy; 3 CNR - Ufficio Agenda Digitale e Processi, Rome, Lazio, 00185, Italy; 4 RDA Professionalising Data Stewardship Interest Group and RDA Data Steward Career Tracks Working Group, Brunico, Trentino Alto-Adige, 39031, Italy; 5 INFN - Laboratori Nazionali del Gran Sasso, Assergi, Abruzzo, 67100, Italy; 6 Politecnico di Torino, Turin, Piedmont, 10129, Italy; 7 Istituto Italiano di Tecnologia, Genoa, Liguria, 16163, Italy; 8 CNR - Istituto di Linguistica Computazionale "Antonio Zampolli", Pisa, Tuscany, 56124, Italy; 9 CNR - Istituto di Ricerca sugli Ecosistemi Terrestri, Lecce, Puglia, 73100, Italy

**Keywords:** survey, data stewardship, Italy, data steward, professional profile, mapping

## Abstract

Research data management (RDM) plays a key role in ensuring research reproducibility and transparency by supporting data sharing in line with Open Science principles. Across the international landscape, professional figures supporting data management practices, such as data stewards (DSs), are emerging through a variety of career paths. The Italian Computing and Data Infrastructure (ICDI), together with the Horizon Europe Skills4EOSC project, conducted a national survey between April and December 2023 to map the presence, roles, and activities of data stewardship services across universities and research-performing organisations. The aim was to provide an initial overview of the Italian landscape, which is known to be fragmented and lacking of clear professional recognition for research support professionals. The survey, administered in Italian through the EUSurvey platform, consisted of 13 closed-ended questions addressing the professional background of DSs, their role and functions, and the structure of RDM support services. Data cleaning was performed on the 77 valid entries collected (out of 88 responses) before conducting the descriptive analyses. Results indicate that most institutions employ staff with DS functions, although these professionals are seldom formally recognised as DSs. The variety of job titles highlighted by the survey reflects the absence of a standardised professional profile. Among the RDM support tasks performed by DSs, the most common include FAIR data management support, data management plan writing, researcher training, and policy consultancy. These findings provided a basis for the establishment of the Italian Data Steward Community, aiming to overcome the fragmentation through peer-to-peer knowledge sharing and support, and to advance the definition of a professionalisation pathway for DSs in Italy. The findings of this survey can serve as a case study to help DSs, research data professionals, and policymakers in other countries to map and understand their local landscape.

## Plain Language summary

Good research depends on data that are well organised, well documented, and easy to share. Managing research data properly helps make research more transparent, trustworthy, and reusable, in line with Open Science principles. To support researchers with data management tasks, new professional roles are emerging. One of these roles is often called *data steward*, although this job title is not used consistently.

This study explores how data management support is organised in Italian universities and research institutions. Between April and December 2023, the Italian Computing and Data Infrastructure, supported by the Horizon Europe Skills4EOSC project, carried out a national survey to understand who provides data management support, what they do, and how they are recognised within their institutions.

The survey included 13 multiple-choice questions and was completed by 88 respondents, of whom 77 were suitable for analysis. The results show that many institutions already have staff members who support researchers with tasks such as writing data management plans, applying FAIR data principles, training researchers, and advising on data policies. However, these professionals often have different job titles and are rarely formally recognised as data stewards.

The study highlights a fragmented landscape and a lack of a clear professional profile in Italy. Based on these findings, the authors propose the creation of an Italian Data Steward Community to encourage knowledge sharing, reduce fragmentation, and support the development of a clear professional pathway. The results may also help other countries better understand and map their own data stewardship landscape.

## Introduction

Data stewards (DSs) are a cornerstone of the research data ecosystem. Born at the intersection of data management, research support, and policy development (policies regarding research data management, Open Science, open access, personal data, knowledge management, etc.), this profession has developed (and continues to develop) in response to the growing complexity of managing, sharing, and preserving digital research outputs. As research institutions have embraced the principles of Open Science and FAIR (Findable, Accessible, Interoperable, and Reusable) ( Wilkinson *et al.*, 2016), the need for dedicated professionals who can bridge the gap between researchers, IT specialists, and policy-makers has become evident. In this article, we consider DSs as key actors in ensuring that research data are not only technically well-managed but also ethically, legally, and contextually sound, and that research-performing institutions have in place policies and workflows that enable FAIR research data management (RDM); DSs can be in advisory roles, or embedded in research groups with more hands-on roles ( The Turing Way Community, 2025). The specific role(s) and function(s) of DSs are not unequivocally defined, and often depend on organisational and national context. The same applies to data stewardship services, i.e. which functions are performed by DSs in a specific context utilising the resources made locally available ( Ayres *et al.*, 2022). For this reason, throughout this article, the terms “DS”, “DS functions” and “data stewardship” are used in a broad sense to refer to all activities related to data stewardship, as well as to all professionals whose roles can be fully or partially associated with this function, acknowledging that these terms may encompass a variety of roles, activities, and organisational interpretations.

This study aims to investigate how data stewardship services are organised in Italian Universities and Research Performing Organisations (RPOs), by applying descriptive statistics to the responses collected from the national DS community via a survey (see Materials and Methods).

The analysis addresses the following research questions:

• RQ1: To what extent are DSs formally integrated in research performing organisations in Italy?
• RQ2: What are the typical training pathways of Italian DSs?
• RQ3: What activities do Italian DSs perform in their organisations?
• RQ4: How are the RDM support services organised in different research performing organisations across Italy?

The article is structured as follows: the introduction provides a brief overview of the emergence of DSs as professional figures in Europe and in Italy; the section ‘Materials and methods’ outlines the methodology; the section ‘Analysis of Survey Results’ presents and discusses the findings; and the section ‘Discussion’ offers final reflections on the findings.

### The emergence of research data stewardship in Europe and beyond

DSs as professionals have emerged only in the last 10 years, at least in research contexts ( Wildgaard *et al.*, 2020). Following the publication of the FAIR principles in 2016 ( Wilkinson *et al.*, 2016), the *Turning FAIR into reality* report by the European Commission’s Expert Group on FAIR Data recognised data stewardship as a key service for enabling FAIR data. The report also highlighted the need for new job profiles in data science and data stewardship to support researchers throughout the research data lifecycle ( European Commission. Directorate General for Research and Innovation, 2018). Around the same time, universities in the Netherlands developed their first dedicated data stewardship programmes, including the 3-year Data Stewardship Project in TU Delft that introduced faculty-level DSs at the university ( Turkyilmaz-van der Velden, 2018). Following the report dissemination, several European countries (e.g. Denmark, Austria, the Netherlands, Germany, and Poland) carried out national-level projects that surveyed local data stewardship landscapes, defined DS roles and service models, identified skills and competencies, and outlined training programmes ( Gruber *et al.*, 2021; Jetten *et al.*, 2021; Seidlmayer *et al.*, 2023; Wałek, 2023; Wildgaard *et al.*, 2020). More recently, a national-level survey has also been completed in Portugal, following the example and adapting the national-level survey in Italy discussed in this article ( Boavida *et al.*, 2025). These national-level projects helped to scope data stewardship in Europe and highlighted similarities and differences in data stewardship service models as well as in DS roles and functions across national contexts.

National-level work in the Netherlands and in Denmark identified three key functional areas for data stewardship: policy, research, and infrastructure, and defined DS roles in these areas ( Schmitz, 2020). The German study identified five DS profiles: (1) generalist, (2) advisory RDM, (3) discipline-specific, (4) coordinating, and (5) infrastructure-focused DS ( Seidlmayer *et al.*, 2023). Although these national-level studies evidence an overlap between DS roles and functions in different national contexts, they also highlight that DS role(s) and function(s) depend on the local context in which data stewardship services are initiated. The latter is confirmed by a recent comparative case study of data stewardship services in universities ( Basalti *et al.*, 2024; Landrgottová, 2026; Rousi, Boehm and Wang, 2024). Moreover, in some (national) contexts the functions of a DS overlap with, or are subsumed by, data librarian or data curator roles ( Ashiq and Warraich, 2023; Federer, 2018; Tammaro *et al.*, 2019). The Austrian study directly links DS roles to their organisational context by introducing DS profiles and related skills and competences through the definition of three data stewardship service models ( Gruber *et al.*, 2021). National-level studies of data stewardship – like the Italian survey presented in this article – contribute to a better understanding of DS roles and how they are carried out in specific organisational contexts, thus supporting a more nuanced understanding of data stewardship services and their development.

### Efforts to professionalise research data stewardship

A better understanding of DSs in various organisational and national contexts also allows for the convergence of DS roles across national, European and international contexts. Several efforts have aimed at aligning DS roles and data stewardship service models. Within the Research Data Alliance (RDA), the Professionalising Data Stewardship Interest Group ^1^ (endorsed in 2020) has completed two global surveys. The first one in 2021 on data stewardship service models ( Ayres *et al.*, 2022) found that most respondents considered their service provision still in the early stages of maturity. A second one, administered in 2022 and focusing on DS career tracks ( Newbold *et al.*, 2024) highlighted that professionals who self-identify as DSs have many different job titles – only 33% of the European respondents in DS roles at the time of the survey (n = 205) actually had the job title “data steward” – and underscored the lack of clearly defined career paths for DSs. An OpenAIRE survey completed in 2023 ( Gurdal and Yavuz, 2023) found that 67% of the responding institutions did not have formal job descriptions for their RDM roles and the professionals in those roles had a variety of job titles (e.g. ‘data steward’, ‘data manager’, ‘project employee’). The work by RDA and OpenAIRE highlights that lack of agreed-upon definitions of DS limits the recognition of DS roles ( Kalová *et al.*, 2024). The Horizon Europe Skills4EOSC project ^2^ built on the DS roles, skills and competences defined in the various national contexts to deliver Minimum Viable Skills profiles for “coordinating data steward” and “embedded data steward” that are intended to have European-level applicability ( Hansen *et al.*, 2025). National-level studies – like the survey results presented in this article – continue to provide valuable input to further define and recognise DS roles at national, European and international level and contribute to the continued professionalisation of data stewardship.

### Data stewardship in Italy

In Italy, similarly to other European countries, the DS role is yet to be formalised. Following the increasing importance of professional RDM activities to support the research lifecycle, several Italian universities and research institutions have progressively introduced data stewardship roles, training opportunities, and governance models. In 2019, a group of research and e-infrastructures in Italy signed the “Italian Computing and Data Infrastructure” (ICDI) *memorandum of understanding.* ICDI is a forum created to promote synergies at the national level and to optimise the Italian participation in European and global challenges in the field of Open Science and FAIR data ( *Italian Computing and Data Infrastructure*, no date). By including several Italian research institutions and universities, ICDI progressively became the forum where Open Science and RDM experts could exchange ideas, start collaborations, and propose solutions to policy-makers with a bottom-up approach. The monthly online “Open Science Cafè” webinars, on major Open Science topics and updates, exemplify ICDI’s efforts in community engagement and building in the Italian landscape ^3^.

One of the leading organisations in ICDI is Consortium GARR ^4^, which also coordinated the aforementioned Horizon Europe Skills4EOSC project. Among its activities, this project defined DS roles and training, and supported the development of professional networks of DSs across Europe. The survey that we discuss in this article emerged from the collaboration between ICDI and Consortium GARR in Skills4EOSC and aimed at understanding better DS roles and services in Italian universities and research-performing organisations. The survey also served as the first step towards developing a national community of practice for DSs and research data management roles. This article presents the survey results, shedding light on DS roles and data stewardship service models in the Italian context. This is the first attempt to study data stewardship in Italy.

## Materials and Methods

### Survey development and distribution

The “Survey on Data Stewards in Italy: a mapping of professionals supporting research data management” was developed as a bottom-up initiative by Consortium GARR, coordinator of the Skills4EOSC project, in collaboration with the University of Bologna (UNIBO) and the Italian Institute of Technology (IIT), under the umbrella of the Italian Computing and Data Infrastructure (ICDI).

The survey was developed as a completely anonymous web-based set of questions using EUSurvey, an open-source platform by the European Commission. It was distributed in Italian to facilitate completion and improve response rates, but an English version was also prepared.

To maximise participation, we initially targeted existing communities interested in RDM topics. We shared the survey via two national mailing lists focused on Open Science (“ oa-italia@lists.icdi.it” and “ icdi@lists.garr.it”), and expanded the audience through word-of-mouth, institutional social media channels (e.g., Skills4EOSC LinkedIn page ^5^), and an article in the GARR Magazine (Colcelli, 2023). We acknowledge the limitations of this approach, as it may have introduced sampling bias (see Discussion section).

The survey was officially launched in April–May 2023 (from 13/04/2023 to 31/05/2023) and reopened in November–December 2023 (from 07/11/2023 to 01/12/2023), aiming to increase participation and expand the geographical coverage. We acknowledge that collecting data during two distinct time periods may represent a limitation of the study, as temporal differences could have influenced respondents’ perceptions and answers.

### Survey and question structure

The introduction of the survey provided a general definition of DS to ensure a shared understanding among respondents, as survey completion was not limited to professionals with the formal job title of DS.

The first section of the survey collected information about the respondents’ organisations and roles. Respondents were also asked to report whether they were completing the survey as individuals in a personal capacity or as representatives of their organisation. This distinction was intended to differentiate responses provided under an official institutional mandate from those based on the respondent’s own knowledge and experience. Regardless of the selected category, respondents reported information about their organisation.

Only those respondents who reported DSs in their organisations could proceed to the second part of the survey; for all others, the survey ended after collecting profile information.

The second section of the survey included 13 closed-ended questions that explored the presence of DSs in Italian research performing organisations, their training pathways, their institutional activities, and the organisation of data stewardship services, with the aim of addressing the research questions presented in the introduction.

To structure data collection and facilitate analysis, the questionnaire consisted mostly of closed-ended questions. We included single-choice questions where respondents were required to select one mutually exclusive option, as well as multiple-choice questions that allowed the selection of more than one response. Open-ended questions were included to capture information that could not be reliably categorised in advance.

We designed the survey to collect only anonymous data from respondents.

The questions were developed through an iterative design process informed primarily by the authors’ direct professional experience, which allowed to capture the context-specific dynamics and practical considerations. This firsthand expertise was complemented by insights drawn from international reports, particularly those developed in the Netherlands and Denmark. As an example, this approach was adopted to compile the list of data stewardship activities presented in the question “What kind of support do the “data stewards” or equivalent figures provide within your organisation?” We archived survey templates, in English and Italian, as well as the survey output dataset, in Zenodo ( Caldoni, Iacovella, *et al.*, 2025).

### Data cleaning and visualisation

A total of 84 anonymous responses were collected and exported as a.csv file, on which data cleaning was performed.

OpenRefine ^6^ () was used to split multiple-choice responses, correct inconsistencies and cluster respondent roles into three macro-categories: “researcher”, “technologist” and “technical-administrative staff”. These three macro-categories were chosen as they represent distinct and formalised functions in Italian universities and research performing organisations. Namely, “researchers” are directly responsible for producing scientific knowledge and conducting original research, while “technologists” provide advanced technical and specialist support to research. This second role typically involves managing laboratories, digital infrastructures, data platforms, scientific instruments, or complex technical workflows. Finally, “technical-administrative staff” ensures the day-to-day functioning of the organisation by handling administrative, financial, and organisational tasks.

Manual data cleaning operations consisted of:

• adding ROR identifiers when not provided by respondents,
• removing responses not from Italy (used for functionality tests),
• adding a column for organisational acronyms,
• classifying organisations (universities vs. research entities),
• eliminating duplicate answers.

Lastly, two responses referring to the affiliated organisation of the respondents were manually inspected and excluded because the authors could unequivocally verify that these records were incorrect.

After cleaning, 77 valid responses remained. The final dataset was reviewed by all authors to ensure consistency and accuracy.

Given the limited sample size, the analysis was restricted to descriptive statistics, performed using Microsoft Excel ^7^.

R was used to produce a map of the geographical distribution of the respondents ( Figure 1). The resulting R-plot was replicated, for visualisation purposes, into a multi-level image using the vector graphics editor Inkscape ^8^. To improve readability, city-centred pie-charts reflecting geographical provenance were subdivided into 4 categories, according to the number of respondents. Cities with a single respondent were chosen as the unit size. Pie charts for cities with 2–5 respondents were visualised as 1.5 times the size of the unit; for cities with 6–14 respondents, the pie chart size was scaled at 2 times the size of the unit. The only pie chart corresponding to a city with more than 14 respondents was resized to 2.5 times the size of the unit.

**Figure 1.**
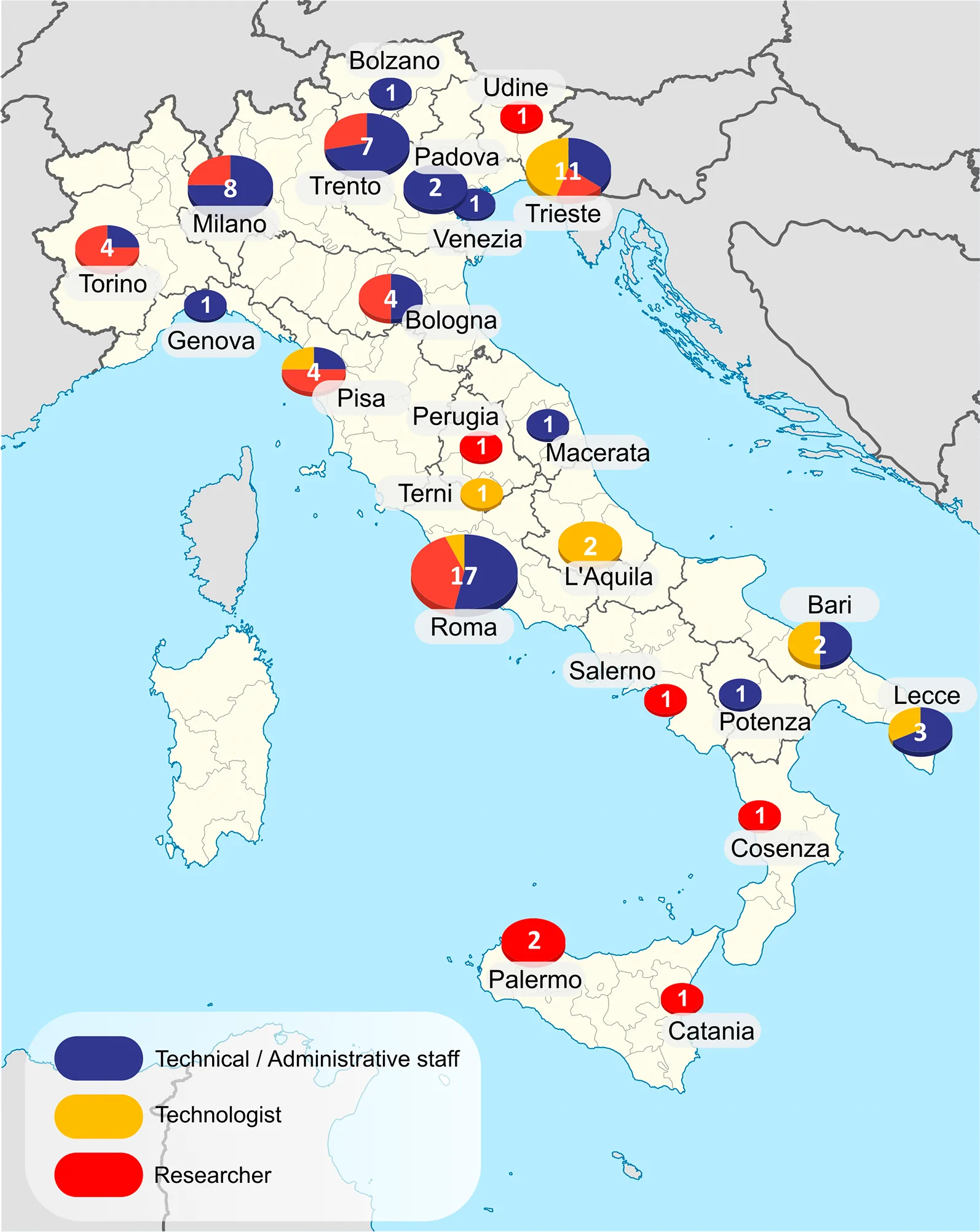
Map of institutional roles represented among respondents to the national survey.

The results of the analysis are presented in graphs, maps and tables to visualise the diversity and geographical coverage of the survey.

## Analysis of Survey Results

### Institutional roles and personal interests

First, we asked respondents to indicate their affiliation and specify their role within the organisation. This granted a better picture of the professionals involved in the study, which was useful in the qualitative analysis of the data.

We collected responses from 48 different institutions (based on unique ROR identifiers), reflecting broad institutional representation. As presented in Figure 1, these institutions are geographically distributed throughout Italy, although regional representation is not uniform.

Out of the total 77 respondents, 33 are affiliated with universities, while 44 are affiliated with public research performing organisations.

Regarding the professional role of the respondents, we considered the three main types of employees within the Italian public research performing organisations: Technical/Administrative staff, Technologist, and Researcher. To highlight possible geographical differences within the respondents’ population, we collated responses from different research performing organisations in the same city. We mounted single-city pie charts on a map of Italian administrative subdivisions ^9^.

The majority of the respondents belong to the Technical/Administrative support staff (n = 38), while another significant portion of respondents identified themselves as a researcher (n = 27). Only a small number of technologists (n = 12) answered the survey, consistent with the uneven distribution of this professional framework across the national landscape (see the section “Data cleaning and visualisation” in the “Materials and Methods” chapter for the definition of these three different professional roles).

As participation was not restricted to individuals formally designated as DSs, the results capture perspectives from a broader community involved in data-related activities. While responses likely reflect participants’ professional experiences, the survey did not explicitly ascertain the perspective from which answers were provided.

Then, we asked whether the respondents were participating in the survey in a personal capacity or whether they were officially representing their organisation. Most of the respondents participated in a personal capacity (57 out of 77, 74%), while 26% responded on behalf of their institution (20 out of 77).

RQ1: To what extent are DSs formally integrated in research performing organisations in Italy?

We started our analysis by examining the presence and the role of DSs or analogous figures in the organisations that participated in the survey. In this section, we focus our analysis solely on the responses provided on behalf of organisations (n = 20).

About 65% (13 out of 20) of the respondents state that there are professionals with DS functions ( Figure 2).

**Figure 2.**
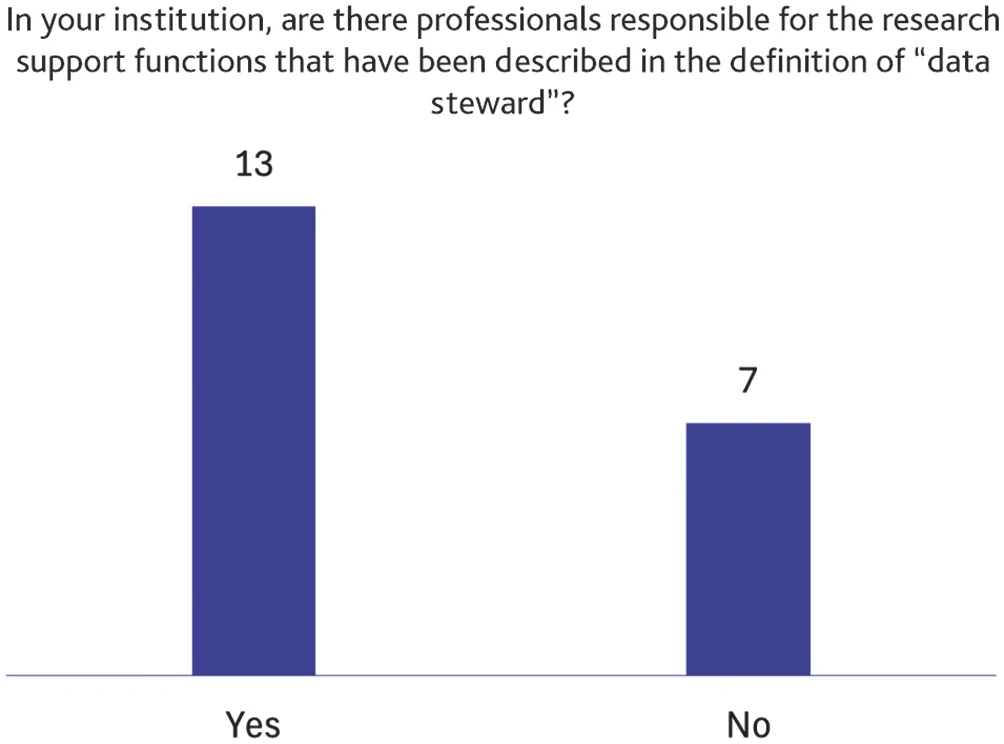
Presence of professional figures performing DS functions. (n = 20 respondents, answering on behalf of their institutions.)

Interestingly, only one out of 13 respondents reported that the DS role is formally recognised ( Figure 3).

**Figure 3.**
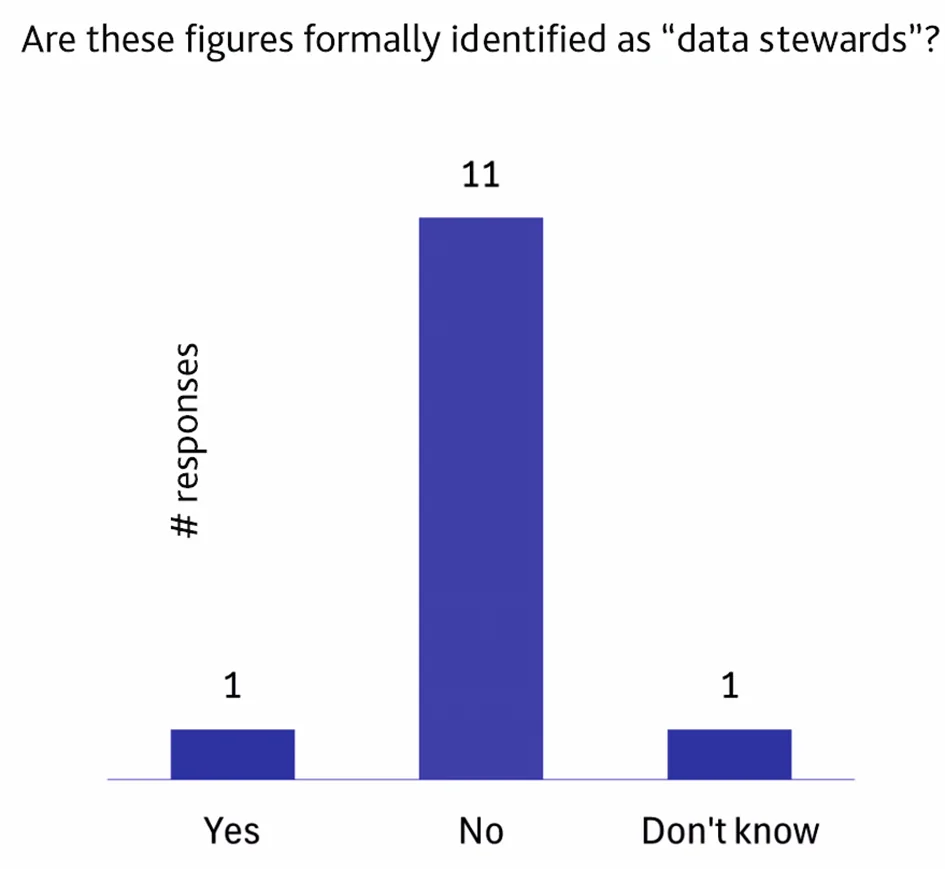
Presence of professional figures supporting research management formally identified as DSs. (n = 13 respondents, answering on behalf of their organisations and reporting that the organisation has staff dedicated to DS functions.)

Table 1 shows that the job titles used to identify professional figures working in DS roles can vary considerably, even within the same organisation. Respondents were allowed to select as many job titles as necessary to describe the reality of data stewardship in their organisation. Respondents were provided with seven possible, non-mutually exclusive response options, partially derived from the roles most represented in the international landscape ( Ayres *et al.*, 2022; Jetten *et al.*, 2021) and had the chance to add more options in a free text box if necessary.

**Table 1.** Breakdown of the formal designation of professional figures with DS functions within organisations.

Job title	Number of responses
Research support staff	2
Data manager	2
Research Data Management Specialist	5
Data scientist	2
Data expert	2
Information specialist	1
Other	3

n = 11 respondents, answering on behalf of their organisations and reporting that the organisation has staff dedicated to DS functions. Multiple (non-mutually exclusive) responses are allowed.

Further evidence of the lack of a clear professional framework for DSs is seen in the distribution of the roles for those performing data stewardship activities, including research roles (post-doc, research fellow, etc.), technical roles (e.g. IT support, data processing technician, etc.) and administrative roles (e.g. librarian, research support, project manager and others). As shown in Table 2, no significant differences were observed across these categories.

RQ2: What are the typical training pathways of Italian DSs?

**Table 2.** Breakdown of the professional roles of DSs within organisations.

Roles	Number of responses
Technical staff	5
Administrative staff	6
Research staff	6
Don’t know	1
Other	2

n = 13 respondents, answering on behalf of their organisations and reporting that the organisation has staff dedicated to DS functions. Multiple (non-mutually exclusive) responses are allowed.

Considering the lack of any officially recognised national training pathway leading to the title of “data steward” in Italy, we asked about the educational background of DSs. For this analysis, we included all respondents who reported the presence of DSs in their organisations (n = 38), without dividing responses given on behalf of the organisation or in a personal capacity. Respondents were allowed to select multiple response options, to allow us to capture the nuances of a single DS training path.

Figure 4 shows the prevalence of specific disciplinary backgrounds. Indeed, the largest group (18 responses) reported a master’s degree or PhD in a relevant disciplinary field (e.g., biology, physics, social sciences). The second largest group (15 responses) indicated degrees in related or complementary fields (e.g., library science, archiving, information science, statistics). A total of 13 respondents reported degrees in Computing or Computer Science. Finally, 8 responses selected “Other” and 6 selected “I don’t know”.

RQ3: What activities do Italian DSs perform in their organisations?

**Figure 4.**
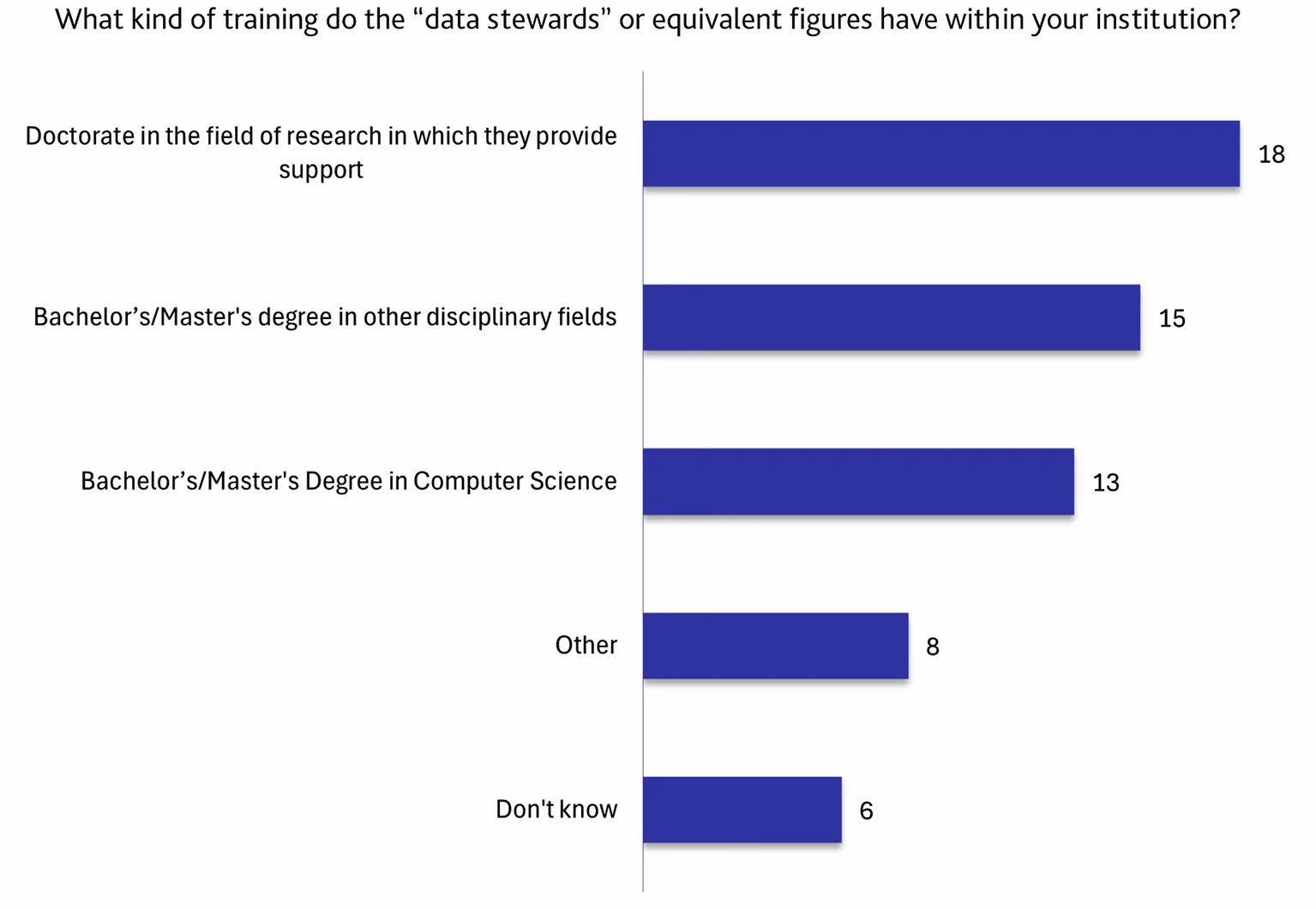
Breakdown of the types of training possessed by DSs before their recruitment within organisations. (n = 38 respondents, answering either on behalf of their organisation or personally, reporting that the organisation has staff dedicated to DS functions. Multiple (non–mutually exclusive) responses are allowed.)

After detailing the DSs’ background, we investigated their activities, focusing mainly on support and data management ( Ayres *et al.*, 2022; Basalti *et al.*, 2024; Jetten *et al.*, 2021). Namely, we asked what kind of activities the DSs perform in their organisation, providing seven possible response options as well as the option to indicate more activities. The list of the response options included:

• “support for research data management and the application of FAIR principles, identification of disciplinary repositories, storage and backup solutions, metadata, etc.;”
• “support for writing the data management plan; assessment of possible legal issues (e.g., privacy, IP and licences, etc.);”
• “drafting of institutional policies for research data management;”
• “advice on national, international and funding body policies and mandates for data management and compliance with any obligations;”
• “training of researchers on Open Science, FAIR data and research data management issues;”
• “support for the use of data management tools made available by their institution (e.g. institutional repository, institutional cloud, etc.);”
• “support for the management and maintenance of the institutional repository (e.g. care and validation of deposited datasets).”

We considered for our analysis here all respondents who indicated that their organisations had DS (n = 38), without restricting our analysis to responses provided on behalf of an institution only. Respondents could select more than one response option to capture the extent to which DS activities are characterised by specialisation or encompass multiple DS functions.

The results ( Figure 5) show that DSs or equivalent figures are prevalently involved in RDM support, particularly in applying FAIR principles (34 responses) and assisting with DMPs (31 responses). A second cluster of activities included drafting institutional policies (24 responses), training on Open Science and RDM (25 responses), and support for institutional data management tools (26 responses). More specialised activities like providing advice on national, international and funding body policies and mandates for data management and compliance with any obligations (19 responses), as well as legal assessment (18 responses) and repository maintenance (18 responses) were less prevalent.

**Figure 5.**
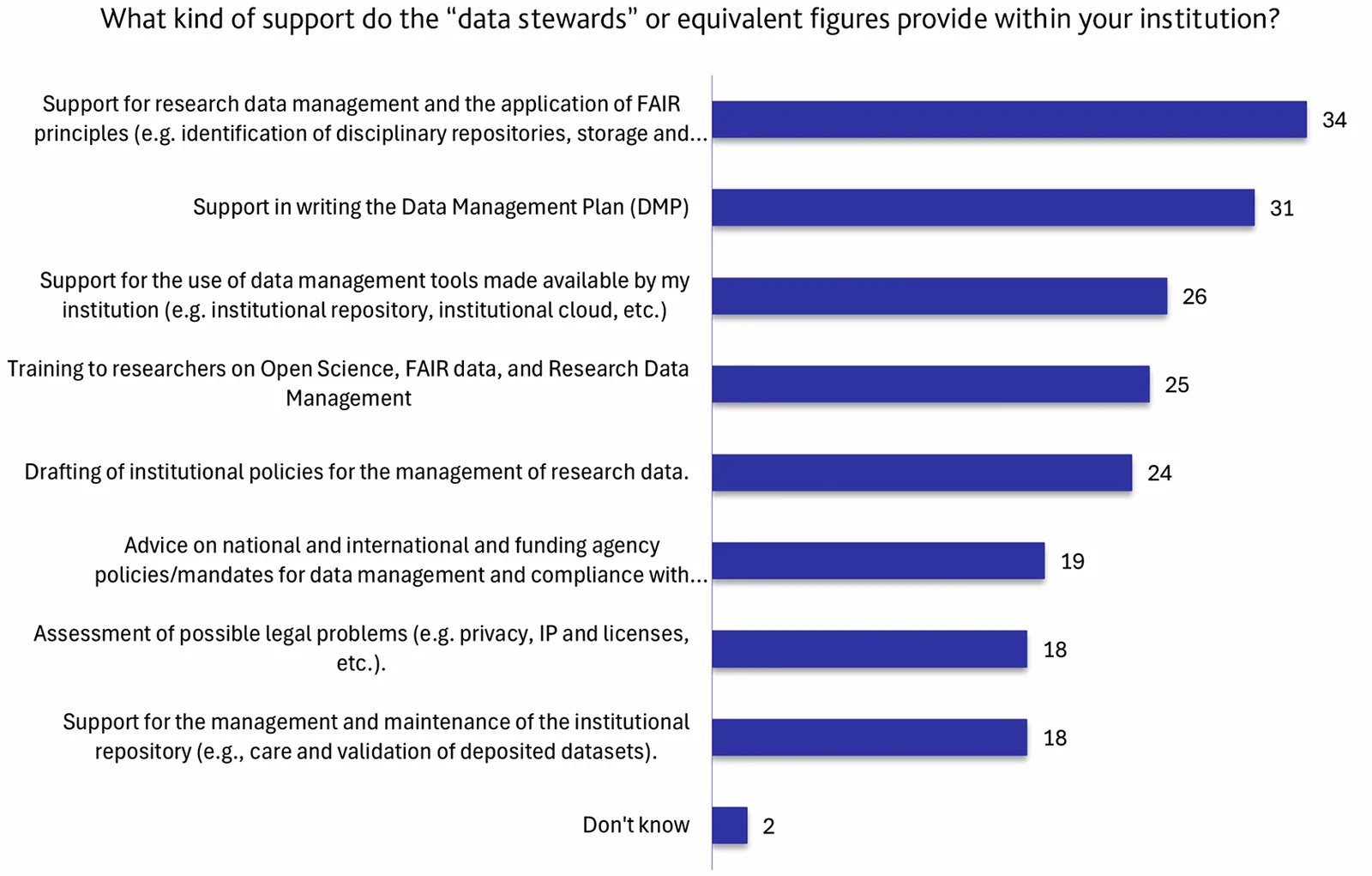
Breakdown of the support functions carried out by data stewards or analogous figures within organisations. (n = 38 respondents, answering either on behalf of their institution or personally, reporting that the organization has staff dedicated to DS functions. Multiple (non–mutually exclusive) responses are allowed.)

As shown in Figure 5, most respondents (95%) selected at least 1 support activity out of the 8 we proposed in the survey. Only 6 respondents (16%) selected 1–3 related activities. 30 out of 38 (more than 75% of the cohort) selected at least 4 activities. This multi-task related partition shows a substantial equality between the number of activities: the number of people selecting 4 to 8 activities is, for all the 5 categories (4,5,6,7,or 8 activities), always about 15% (respectively, 6,5,5,7, and 7 people). By focusing on the 11 (about one third of the cohort) respondents selecting 4–5 activities, there is a solid pattern (10 out of 11) of selecting both RDM and DMP. This often (7 people) occurs together with training duties. A substantial proportion of respondents of the entire cohort (19 out of 38, 50%) selected 6 or more different activities. They covered most of the proposed activities, with slightly fewer mentions (13 out of 19) of the “Advice on local and international policies” and the “Institutional repository” activities.

After mapping the type of activities provided, we decided to investigate the target groups of these services. In particular, we asked whether these services were only available to researchers with project-related data management obligations. 29 (out of 38) respondents (76%) indicated that services are available to all research staff, regardless of project obligations.

RQ4: How are the RDM support services organised in different research performing organisations across Italy?

In the last RQ, we proceeded to investigate how DS activities are organised. We based the analysis on 38 out of 77 respondents indicating the presence of DSs in their organisations.

As shown in Table 3, most respondents (26 out of 38) reported that their organisation had two or more professionals performing roles equivalent to DSs. The presence of a single individual in this role was reported in seven organisations.

**Table 3.** Count of DSs within organisations.

Number of DS or equivalent figures	Number of responses
Don’t know	5
N > 10	2
2 ≤ N ≤ 10	24
N = 1	7

N = number of DSs; n = 38 respondents, answering either on behalf of their organisations or in a personal capacity, reporting that the organisation has staff dedicated to DS functions.

Figure 6 shows that in 42% of cases (16 out of 38 responses) DSs are embedded within research facilities, while 26% (10 out of 38) frame these professional figures within central administrative areas. Interestingly, 21% (8 out of 38) of respondents reported a hybrid situation, with DS roles established at both levels.

**Figure 6.**
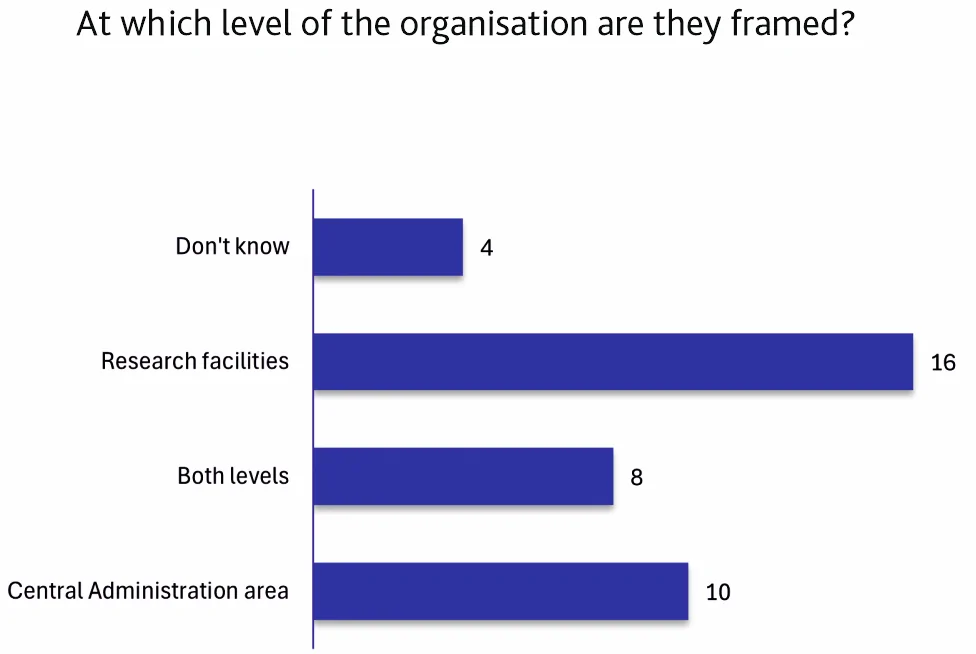
Level of the organisation where “data stewards” or equivalent figures are framed within organisations. (n = 38 respondents, answering either on behalf of their organisation or personally, reporting that the organisation has staff dedicated to DS functions.)

To further characterise the organisational placement of DSs, we combined responses indicating a central administrative location with those reporting a hybrid model, resulting in a total of 18 cases. The distribution of these cases across specific central organisational units is presented in Table 4.

**Table 4.** Breakdown of the central administration areas where DSs are framed within organisations.

Central administration area: services or units involved	Number of responses
Research support	12
Research organisation	4
IT	4
Library	1
Don’t know	2
Other	2

n = 18 respondents, answering either on behalf of their organisations or personally, reporting that the organisation has staff dedicated to DS functions and employed, either exclusively or partially, in a central administration area. Multiple (non–mutually exclusive) responses are allowed.

Since this question allowed for multiple, non-mutually exclusive responses, three out of 18 respondents indicated more than one area of the central administration, in particular, research support, IT, and administrative divisions. This may reflect the interdisciplinary and cross-functional nature of the DS role, even within centralised structures.

## Discussion

The findings of this survey provide an overview of the emerging professional landscape of data stewardship in Italian universities and public research performing organisations.

We observed that only a minority of respondents participated in the survey on behalf of their organisation. Consistent with the European trends reporting a correlation between institutional maturity in RDM and the adoption of DS roles ( Gruber *et al.*, 2021; Jetten *et al.*, 2021; Seidlmayer *et al.*, 2023; Wildgaard *et al.*, 2020) it is reasonable to infer that organisations with higher levels of awareness and engagement in RDM are more likely to be developing or implementing structured RDM support services and to have designated a representative for survey completion.

The results collected with RQ1 indicate that the DS role is increasingly recognised as essential in the research landscape, yet still characterised by heterogeneous role definitions and limited institutional recognition. We decided to consider only responses provided on behalf of the different organisations (n = 20) as our interest was focused on capturing the formal and institutional definition of the DS role.

We observed that approximately 65% (13 out of 20) of the respondents reported the presence of professionals performing DS functions ( Figure 2). As shown in Figure 3, the DS role is formally recognised only in one case. On the other hand, we observed a variety of job titles associated with DS roles, even within the same organisation ( Table 1). Taken together, these findings show that research performing organisations in Italy recognise the relevance of introducing DS functions (e.g. support to RDM and DMP drafting/updating, promote the adoption of FAIR principles, facilitate data sharing in compliance with ethical, legal, and security requirements, training, etc.). However, this awareness does not lead to the formal recognition or standardisation of DS role(s), a finding which aligns with the international landscape ( Ayres *et al.*, 2022; Hansen *et al.*, 2025; Newbold *et al.*, 2024). The fact that DSs appear to be equally employed either as technical, administrative or research staff ( Table 2) further builds on these observations, suggesting that DS functions are often assigned to previously existing roles, without providing adequate formalisation, visibility, or clear career pathways.

We also note that 35% of the respondents (7 out of 20, Figure 2) reported the absence of DSs in their organisation, suggesting uneven levels of awareness on this topic.

Hence, and in line with previous surveys in other national contexts ( Jetten *et al.*, 2021; Seidlmayer *et al.*, 2023; Wildgaard *et al.*, 2020), our results show that the definition of the DS role depends heavily on the organisation, underlining the absence of a coordinated national framework.

With RQ2 and RQ3, we aimed to characterise the professional identity of DSs, specifically in terms of their educational background and the activities they perform. In the majority of cases, DSs hold advanced degrees in disciplinary fields related to the scientific discipline in which they provide data stewardship ( Figure 4). Domain-specific expertise is a key asset to provide effective data stewardship services, as it enables DSs to understand disciplinary research practices and facilitates communication with researchers ( Gruber *et al.*, 2021; Jetten *et al.*, 2021; Seidlmayer *et al.*, 2023; Wildgaard *et al.*, 2020). Interestingly, an educational background in computer science is frequently reported, thus suggesting a growing emphasis on the technical dimension of the DS role. Nonetheless, the results reflect a lack of consensus regarding the educational pathways necessary for DS role.

When looking at DS functions, according to the responses gathered in Figure 5, a DS is a professional with at least one or more cross-cutting skills in RDM (e.g. disciplinary, IT and technical, legal). Among the eight activities included in the survey, support for RDM and for DMP drafting are the most frequently reported. Interestingly, these core activities are often carried out alongside two or more additional activities, such as support for the use of RDM tools provided by the organisation, training and capacity-building efforts. These results highlight that a single DS should be able to perform multiple DS functions, which fall between those specific to the research domain and those typical of administrative functions.

Overall, these findings depict a complex landscape in which DSs are expected to acquire and develop a broad range of skills to perform effectively, while the corresponding training pathways remain unclear and insufficiently aligned, often relying on traditional learning models that do not fully address all relevant skills and competencies. This gap highlights the need for more structured, comprehensive, and role-specific training frameworks to better align professional preparation with the evolving demands of the field.

The final part of our survey aimed to portray how DS services are organised. Most respondents reported that their organisation employs between two and ten professionals performing the DS role (24 out of 38 respondents, Table 3). While the range is too broad to accurately characterise the typical number of DSs in research performing organisations, it suggests that the preferred strategy is to employ more than one person performing such functions. The second most common response indicates the presence of only one DS in the organisation, showing different approaches to the delivery of data stewardship services in different research performing organisations.

This diversity is further sustained when investigating at which level DSs are introduced in research performing organisations ( Figure 6): according to our results, we observe that DSs are more frequently employed in research facilities (n = 16) than in central administration (n = 10), with 8 cases reporting their presence at both levels. This distribution is coherent with the description of two different DS profiles presented by the Skills4EOSC Minimum Viable Skills profiles ( Hansen *et al.*, 2025). Specifically, the project identified the “Coordinator” DS as the one providing support across an organisation’s research domains and units, while the “Embedded” DS operates close to a research team and to its domain-specific practices. The data collected with our survey seem to support the presence of these different profiles in Italy as well. Interestingly, around 20% of the respondents (8 out of 38) reported that DS are framed at both levels, suggesting that some organisations are still experimenting with hybrid service models that can benefit the general academic community while also providing specialised support.

Overall, this study provides initial insights into the evolution and development of the DS role in Italian research performing organisations. The findings suggest a growing recognition of DS functions and their gradual, though heterogeneous, integration into institutional practices and service frameworks, which appear to be diversified. The DS role is intrinsically interdisciplinary, combining research, technical and support competencies, yet the results highlight the need for clearer role definition, more structured training and stronger national coordination.

We acknowledge the limitations of this analysis. First, the analysis is based on data collected in 2023 and therefore may not fully reflect the current state of the Italian DS landscape. Second, the dissemination strategy reflected the bottom-up nature of the initiative and relied primarily on channels associated with the RDM communities. While this approach was effective in reaching professionals likely to be involved in data stewardship activities, it may also have introduced a sampling bias, potentially overrepresenting research performing organisations with established RDM services. Finally, although the survey allowed us to gather responses from across the country ( Figure 1), we recognise that they represent only a limited fraction of Italian research performing organisations, with responses obtained from 17 out of the 104 universities and research centres operating in Italy ^10^. This should be considered in light of the study’s primary aim, which was to provide a snapshot of the existing practices and roles rather than to develop a comprehensive representation of the entire national context. A further consideration is that participation was open to professionals with diverse roles and was not restricted to individuals formally serving as data stewards. Consequently, the findings reflect perspectives from a broader data stewardship community. While respondents likely drew on their own professional experiences, the survey did not explicitly determine whether answers referred to personal practice or broader organizational observations.

Despite these limitations, our findings offer a starting point to support institutional decision-makers not only in defining and structuring data stewardship services within an increasing number of universities and research performing organisations, but also in advancing the professionalisation of the DS role and the development of dedicated career paths. Beyond its immediate results, the survey represents a valuable output in itself, as its methodology can be readily adopted in other national contexts seeking to undertake a similar landscape analysis. This has been recently demonstrated in Portugal ( Boavida *et al.*, 2025).

Moreover, the survey served as the initial step to kick off the Italian Data Stewards Community (Comunità Italiana Data Steward, CIDS) in 2023. The Community has since articulated three overarching objectives, formalised in its 2025 manifesto ( Caldoni, Coppini, *et al.*, 2025), which are aligned with the main observations reported in this study: (i) to create opportunities for sharing and developing new skills among DSs; (ii) to support the adoption of best practices in RDM; and (iii) to increase awareness of the DS’s role within the research ecosystem. As a bottom-up initiative, the Community now includes more than 150 members and promotes networking and knowledge exchange through regular meetings, publications, and collaborative activities. The survey thus played a foundational role in fostering the Community’s self-recognition and professional consolidation within the Italian context.

## Data Availability

Zenodo: Data and code for Mapping Data Stewardship in Italy: Findings from the First National Survey (1.0). https://doi.org/10.5281/zenodo.17907703 ( Caldoni, Iacovella, *et al.*, 2025). The project contains the following underlying data: Data_Figure1.csv (Portion of the whole dataset, integrated with geographical information related to the institutions of the respondents, ready to be used by “Make_Figure1.R script). Data_Survey.csv (File containing the whole dataset acquired through the survey). DS_Survey_Template_EN.pdf (English version of the text of the survey presented to the participants). DS_Survey_Template_IT.pdf (File containing the text of the survey presented to the participants). Make_Figure1.R (R code to produce Figure 1 on “Mapping Data Stewardship in Italy: Findings from the First National Survey” manuscript). Readme.pdf (Readme file describing the content of the dataset).

## Footnotes

1 RDA Professionalising Data Stewardship Interest Group, https://www.rd-alliance.org/groups/professionalising-data-stewardship-ig/activity/, URL checked on 2025/11/28

2 Skills for the European Open Science Commons: creating a training ecosystem for Open and FAIR science, https://www.skills4eosc.eu/, URL checked on 2025/12/04

3
https://www.icdi.it/it/attivita/competence-centre/open-science-cafe

4
https://www.garr.it/it

5
https://www.linkedin.com/company/skills4eosc/, URL checked on 2025/12/11

6 OpenRefine, v3.8.7, https://openrefine.org/, URL checked on 2026/07/21

7 Microsoft ^®^ Excel ^®^ per Microsoft 365 MSO (Version 2606 Build 16.0.20131.20154, 64 bit)

8 Inkscape 1.3 (0e150ed6c4, 2023-07-21), https://inkscape.org, URL checked on 2025-24-10

9
https://commons.wikimedia.org/wiki/File:Italy_provincial_location_map_2016.svg, URL checked on 2025/10/24

10
https://www.mur.gov.it/it/aree-tematiche/universita/le-universita , URL checked on 2025/12/02 https://www.mur.gov.it/it/aree-tematiche/ricerca/il-sistema-della-ricerca/enti-di-ricerca-pubblici , URL checked on 2025/12/02
