# Supplementary material for: Data stewardship in Italy: Findings from the first national survey
Source: Open Res Eur. 2026 Jul 31;6:45. Originally published 2026 Feb 13. [Version 2] doi: 10.12688/openreseurope.22183.2 (PMC12972714; doi:10.12688/openreseurope.22183.2)
Supplement: Peer review history

# Reviewer report: Reviewer response for version 2

**DOI:** 10.21956/openreseurope.26746.r78363

**Published:** 2026-09-07

**Authors:** Antti M. Rousi

**Affiliations:** 1 Aalto University, Espoo, Espoo, Finland

Dear Authors,

 Thank you for the revised version. I genuinely appreciate the amount of work you put into the revision. Thank you also for the detailed replies to reviewers. The well-crafted replies signal that you value our work. Despite major improvements, I think the paper still needs to be finalized for better clarity before it can be indexed. Please see my detailed suggestions below. I hope the authors find my comments helpful.

 General remarks

 Although the authors have put much effort into revising the article, the current revision still displays carelessness in the form of, e.g., discrepancies between reply to reviewers and article execution and in my understanding misplaced Tables. The authors need to still review the text thoroughly for more consistent terminology and presentation.  

 In my understanding, the findings could be summarized as “There is a growing recognition of the importance of data stewardship among Italian universities and research institutions. However, there is no common professional profile for the personnel currently undertaking data stewardship activities in Italian research organizations. Moreover, the professional backgrounds of these personnel vary, which highlights the need for standardized training and clearer career paths for data stewards. The authors suggest a stronger national coordination to develop trainings, more standardized professional profiles, and career paths for data stewards in Italian research organizations.”  

 The above findings are often clouded in too general or vague terms, for instance, the authors seem to use the term “organizations” and “Italian research organizations” interchangeably. The authors should use the term “organizations” whenever they wish to make generalizations about research performing organizations in general and “Italian research organizations” when they generalize about Italian research organizations, and “participating organizations” or “participating Italian organizations” when they speak about the collected data (also “research landscape” vs. “Italian research landscape” and “fragmentation” vs. “no unified professional profile”?). The authors should carefully revise the entire manuscript to provide a clearer terminology as suggested above, esp. the Abstract and Discussion segments.

 There are many one or two sentence paragraphs. In my opinion, article texts in high-quality scientific journals are grouped into larger paragraphs, facilitating understanding and making the reading experience better. The authors should review the entire text and see if they can group the many one or two sentence paragraphs into meaningful larger paragraphs, esp. Data cleaning and visualization, Analysis of survey results, Discussion segments.

 The sentences can be long and involve several complex ideas. Try also to divide the most condensed broad sentences into perhaps two or three sentences for better readability.  

 Cross-check all numbers presented in text so that they correspond with the numbers in Tables and Figures. Then, carefully check that all numerical results in text, Tables and Figures correspond with the published data.

 Title and abstract

 There is a discrepancy between an answer given to reviewers and the papers execution.

 A title “Data stewardship in Italy: Findings from a national survey” was previously suggested by a reviewer, and the authors claimed to accept this suggestion (“hence we accepted the suggested form “Data stewardship in Italy: Findings from a national survey”) However, the current execution of the title is “Data stewardship in Italy: Findings from the first national survey”. I still suggest the authors to consider wording the title as “Data stewardship in Italy: Findings from a national survey” as the use of “first national survey” can be problematic.

 Analysis of survey results

 I believe Table 2 and Figure 4 are misplaced. I believe Table 2 should be presented before going into the RQ2 segment in the findings segment. Also, I believe Figure 4 should be presented before going into the RQ3 segment.

 When discussing the results to RQ3 the authors first list the response options within the survey “The list of the response options included: […]” in the text leading to Figure 5. I find this unnecassary. These options are presented in Figure 5. I suggest the authors to find a work-around for this. 

 Discussion

 Discussion – 2 ^nd^ paragraph

 “We observed that only a minority of respondents participated in the survey on behalf of their organisation. Consistent with the European trends reporting a correlation between institutional maturity in RDM and the adoption of DS roles ( Gruber *et al.*, 2021; Jetten *et al.*, 2021; Seidlmayer *et al.*, 2023; Wildgaard *et al.*, 2020) it is reasonable to infer that organisations with higher levels of awareness and engagement in RDM are more likely to be developing or implementing structured RDM support services and to have designated a representative for survey completion.” I do not understand the relation between these two ideas? Can the RDM maturity level of an organization really be inferred based on whether the respondent saw themselves as being able to respond on behalf of the organization? And if so, do the authors then mean that the Italian ROs are currently at low-maturity levels with regards to RDM? I think the latter claim would need more discussion and findings behind it – could the authors explain and expand?   

 I recommend presenting a concluding Conclusion paragraph or segment to end the Discussion segment, after the limitations. The current concluding content is in different places and is difficult to read and understand (“Overall, this study provides initial insights into the evolution and development of the DS role in Italian research performing organisations. The findings suggest a growing recognition of DS functions and their gradual, though heterogeneous, integration into institutional practices and service frameworks, which appear to be diversified. The DS role is intrinsically interdisciplinary, combining research, technical and support competencies, yet the results highlight the need for clearer role definition, more structured training and stronger national coordination.”). Please divide the content into separate sentences, expand your concluding discussion, and perhaps provide a separate Conclusion segment based on these findings (see also general comments about presenting the main findings more clearly)

 References

 I appreciate that the authors broadened their literature review. However, there are still some prominent studies missing from their list of references (see examples below). It is on the authors’ ethical responsibility to cite the prior relevant literature and therefore it would be important to still search for and expand the list of referenced literature.

 Wendelborn, C., Anger, M., & Schickhardt, C. (2023). What is data stewardship? Towards a comprehensive understanding. *Journal of Biomedical Informatics*, *140*, 104337.

 Fitsilis, P., Damasiotis, V., Dervenis, C., Kyriatzis, V., & Tsoutsa, P. (2024). Effective data stewardship in higher education: Skills, competences, and the emerging role of open data stewards. *arXiv preprint arXiv:2410.20361*.

 Mons, B. (2018). *Data stewardship for open science: Implementing FAIR principles*. CRC Press.

If applicable, is the statistical analysis and its interpretation appropriate?

Not applicable

Is the study design appropriate and is the work technically sound?

Yes

Is the work clearly and accurately presented and does it engage with the current literature?

Partly

Are the conclusions drawn adequately supported by the results?

Partly

Are sufficient details of methods and analysis provided to allow replication by others?

Yes

Are all the source data and materials underlying the results available?

Yes

Reviewer Expertise:

Data stewardship, Information science, scientific communication

I confirm that I have read this submission and believe that I have an appropriate level of expertise to confirm that it is of an acceptable scientific standard, however I have significant reservations, as outlined above.

# Reviewer report: Reviewer response for version 1

**DOI:** 10.21956/openreseurope.24014.r69758

**Published:** 2026-03-12

**Authors:** Samsul Farid Samsuddin

**Affiliations:** 1 Universiti Malaya, Kuala Lumpur, Kuala Lumpur, Malaysia

Overall, it is a good study that have been conducted. However, as the authors aware and concerns, the findings of the study cannot generalize the whole country findings. It was only small portion of institution involved. The sampling approach (snowball sampling and followed approach) are not suitable for this specific study. The sampling should be conducted in more concise so that all related institutions will be much involved and not left behind, and the authors could find a better finding represent the current objective of the study. Dealing with online responses, always across the common method bias issues. Providing how the authors overcoming the issues will much strengthen the responses gathered throughout the study. The findings of data analysis also a bit confusing. Inconsistent numbers of response being analyzed throughout the study. Also not involving the whole 77 responses without any concrete justifications. Which in the earlier part, the study only involved responses representing their institution and not involving the responses as an individual which are the bigger portion. Not providing any justifications on each decision made on the analyses. The discussion part was very comprehensive and in-depth in contributing to the body of knowledge. Much to be improved if the manuscript to be indexed. Current manuscript not ready to be indexed which it will caused misunderstandings and confused to the readers. Details of comments are as follows:

 1. Objectives

1. Map the state of data stewardship in Italian universities and research-performing institutions.
2. A further objective of the survey was to assess the respondents’ willingness to join a national community of practice on RDM.

- The objective of the study not clear. Should detailed out what the authors meant on the state.
- Providing clear several research questions will help readers to relate with all the analysis conducted and findings interpretation.

2. There were no theoretical frameworks or underpinning theories involved in this study seems a bit loose.

 3. Materials and methods

 3.1 The survey was officially launched in April–May 2023 (from 13/04/2023 to 31/05/2023) and reopened in November–December 2023 (from 07/11/2023 to 01/12/2023).

- No explanations or justification why it was reopened.

3.2 Institutional Characterization: Included 13 closed-ended questions to explore how the DS role is defined and how related services are structured in the respondent’s institution.

- Not stated how the questions were developed or not based on any developed questionnaire.

3.3 Abstract – Data cleaning was performed on the 77 valid entries collected (out of 88 responses) before conducting the descriptive analyses.

 Data cleaning and visualization (in content) – A total of 84 anonymous responses were collected and

 exported as a .csv file, on which data cleaning was performed.

- The number of responses is not consistent. 88 or 84 responses?

3.4 Dissemination followed a snowball sampling approach, beginning with targeted outreach, through two national mailing lists focused on Open Science, and expanding the audience through word-of-mouth, institutional social media channels, and an article in GARR Magazine.

- The dissemination approach of the survey was not compatible with the objective of the study. To map the state of data stewardship in Italian universities and research-performing institutions, a strategic and systematic approach should be conducted at the first place. List out all the Italian universities and research-performing institutions and contacted the related departments or units will benefit the study. The findings will be better and it will help future research to be conducted in clearer path with comprehensive findings.

4. Analysis of survey results

 The majority of the respondents belong to the Technical/Administrative support staff (n=38), while another important portion of respondents identified themselves as a Researcher (n=27). Only a small number of Technologists (n=8) answered the survey,

- The total number according to the data reported is 73 responses. Not similar with earlier claim on 77 valid entries collected.
- The authors did not stated the Italian universities and research-performing institutions involved in the study. This might help future scholars to understand the current state.

5. Authors should consider to have a separated sub-heading of Conclusion section for the two last paragraphs.

 6. Ethics and consent: Authors stated that “Ethical approval and consent were not required for this submission.” There was no further justification on this statement. What is the reason of such study did not require any ethical approval and consent when it involved human response. A lot of potential harms might affect when the response was collected, especially when it’s on behalf of the institution and the privacy of the respondents.

If applicable, is the statistical analysis and its interpretation appropriate?

Partly

Is the study design appropriate and is the work technically sound?

Partly

Is the work clearly and accurately presented and does it engage with the current literature?

Yes

Are the conclusions drawn adequately supported by the results?

Yes

Are sufficient details of methods and analysis provided to allow replication by others?

No

Are all the source data and materials underlying the results available?

Yes

Reviewer Expertise:

Areas of expertise on quantitative research and information science fields (including information and research data management aspects).

I confirm that I have read this submission and believe that I have an appropriate level of expertise to state that I do not consider it to be of an acceptable scientific standard, for reasons outlined above.

# Response

**Published:** 2026-07-22

**Authors:** Giulia Caldoni

**Affiliations:** University of Bologna, Bologna, Emilia-Romagna, Italy

*We thank the reviewer for the careful assessment of our manuscript and for the valuable comments and suggestions. Their feedback helped us improve the clarity of the text and provide further explanation on several aspects that required additional attention.* *Below, we provide a detailed point-by-point response to each comment. Our responses follow each of the reviewer’s comments.*

 Overall, it is a good study that have been conducted. However, as the authors aware and concerns, the findings of the study cannot generalize the whole country findings. It was only small portion of institution involved. The sampling approach (snowball sampling and followed approach) are not suitable for this specific study. The sampling should be conducted in more concise so that all related institutions will be much involved and not left behind, and the authors could find a better finding represent the current objective of the study.

 Dealing with online responses, always across the common method bias issues. Providing how the authors overcoming the issues will much strengthen the responses gathered throughout the study. The findings of data analysis also a bit confusing. Inconsistent numbers of response being analyzed throughout the study. Also not involving the whole 77 responses without any concrete justifications. Which in the earlier part, the study only involved responses representing their institution and not involving the responses as an individual which are the bigger portion. Not providing any justifications on each decision made on the analyses. The discussion part was very comprehensive and in-depth in contributing to the body of knowledge. Much to be improved if the manuscript to be indexed.

 Current manuscript not ready to be indexed which it will caused misunderstandings and confused to the readers. Details of comments are as follows: 1. Objectives

1. Map the state of data stewardship in Italian universities and research-performing institutions.
2. A further objective of the survey was to assess the respondents’ willingness to join a national community of practice on RDM.

- The objective of the study not clear. Should detailed out what the authors meant on the state.
- Providing clear several research questions will help readers to relate with all the analysis conducted and findings interpretation.

**Authors’ response:** We thank the reviewer for the observation. We defined 4 Research Questions (RQs) in the *Introduction *section. We have revised the *Analysis of Survey Results* section to organise more clearly the presentation of the findings around the four RQs, ensuring that each RQ is clearly addressed in a dedicated subsection. Furthermore, we have revised the *Discussion* section to summarise more explicitly the key findings in relation to each RQ.

 2. There were no theoretical frameworks or underpinning theories involved in this study seems a bit loose.

**Authors’ response:** We thank the reviewer for the comment. We made the rationale more explicit in the *Materials and Methods* section. The authors’ direct professional experience was complemented by insights drawn from international reports, allowing to transfer international experiences to the Italian context.

 3. Materials and methods 3.1 The survey was officially launched in April–May 2023 (from 13/04/2023 to 31/05/2023) and reopened in November–December 2023 (from 07/11/2023 to 01/12/2023).

- No explanations or justification why it was reopened.

**Authors’ response:** We thank the reviewer for the comment. To improve clarity, we revised the *Materials and methods* section to provide more details on the reasons for reopening the survey. We acknowledged and discussed potential limitations of the dissemination approach, including the survey being reopened during two separate periods, in the *Discussion* section. 3.2 Institutional Characterization: Included 13 closed-ended questions to explore how the DS role is defined and how related services are structured in the respondent’s institution.

- Not stated how the questions were developed or not based on any developed questionnaire.

**Authors’ response:** We thank the reviewer for the comment. The development of the questionnaire has been explained in the final paragraph of the "Survey and question structure" section of the *Materials and Methods*. 3.3 Abstract – Data cleaning was performed on the 77 valid entries collected (out of 88 responses) before conducting the descriptive analyses. Data cleaning and visualization (in content) – A total of 84 anonymous responses were collected and exported as a .csv file, on which data cleaning was performed.

- The number of responses is not consistent. 88 or 84 responses?

**Authors’ response:** We thank the reviewer for pointing out the issue. 88 is the correct number, we identified and corrected a typo.

 3.4 Dissemination followed a snowball sampling approach, beginning with targeted outreach, through two national mailing lists focused on Open Science, and expanding the audience through word-of-mouth, institutional social media channels, and an article in GARR Magazine.

- The dissemination approach of the survey was not compatible with the objective of the study. To map the state of data stewardship in Italian universities and research-performing institutions, a strategic and systematic approach should be conducted at the first place. List out all the Italian universities and research-performing institutions and contacted the related departments or units will benefit the study. The findings will be better and it will help future research to be conducted in clearer path with comprehensive findings.

**Authors’ response:** We thank the reviewer for this important comment. We agree that a systematic census-based approach targeting all Italian universities and research-performing institutions could provide a more comprehensive institutional mapping. However, our study was conceived as a bottom-up community-driven initiative, aimed at obtaining an initial overview of the emerging data stewardship landscape in Italy while simultaneously engaging practitioners and stakeholders active in the field. To avoid misunderstandings, we have clarified this aspect in the manuscript, explicitly stating that the survey dissemination strategy was designed to reach individuals and institutions through existing professional networks and communities of practice, rather than through a formal institution-by-institution sampling process. We acknowledge this as a limitation of the study and note that future research could build on our findings by adopting a more systematic and targeted institutional approach.

 4. Analysis of survey results The majority of the respondents belong to the Technical/Administrative support staff (n=38), while another important portion of respondents identified themselves as a Researcher (n=27). Only a small number of Technologists (n=8) answered the survey,

- The total number according to the data reported is 73 responses. Not similar with earlier claim on 77 valid entries collected.

**Authors’ response:**

- We thank the reviewer for pointing out the issue. 77 is the correct number (Technologists n=12), we identified and corrected a typo.
- The authors did not stated the Italian universities and research-performing institutions involved in the study. This might help future scholars to understand the current state.

**Authors’ response:** We thank the reviewer for this comment. Details about the Italian universities and research-performing institutions involved in the study, are available in the dataset *Data_survey.csv* which has been deposited on Zenodo ( https://doi.org/10.5281/zenodo.17907703).

 5. Authors should consider to have a separated sub-heading of Conclusion section for the two last paragraphs.

**Authors’ response: ** We thank the reviewer for the comment. We thoroughly revised the Discussion section to make it clearer. We decided not to divide it into sections as we believe that it would have hindered the readability of the paragraph.

 6. Ethics and consent: Authors stated that “Ethical approval and consent were not required for this submission.” There was no further justification on this statement. What is the reason of such study did not require any ethical approval and consent when it involved human response. A lot of potential harms might affect when the response was collected, especially when it’s on behalf of the institution and the privacy of the respondents.

**Authors’ response: ** We thank the reviewer for highlighting this important issue. We have revised the Ethics Statement to clarify the rationale for not requiring formal ethical approval and individual written consent. First, the survey was anonymous. Participants were informed in the survey introduction that: “The survey is anonymous and therefore none of the answers will be related to the participant's personal data.” No personal identifiers were collected, and no responses could be linked to individual respondents. Second, the questionnaire did not collect sensitive personal information, nor did it include questions regarding health status, political or religious beliefs, personal behaviors, psychological traits, or other topics that could expose participants to personal, social, professional, or legal risks. The survey focused exclusively on institutional aspects related to the presence, role, training, and activities of data stewards and similar research-support professionals within Italian universities and research institutions. Participation was voluntary, and respondents were informed about the purpose of the study before completing the questionnaire. By choosing to complete and submit the survey, participants provided their informed consent to participate.

# Reviewer report: Reviewer response for version 1

**DOI:** 10.21956/openreseurope.24014.r69762

**Published:** 2026-03-10

**Authors:** Jan Slifka

**Affiliations:** 1 Czech Technical University in Prague, Prague, Prague, Czech Republic

This article presents a valuable first national survey of data stewardship in Italian universities and public research institutions. The topic is timely and relevant, the manuscript is well situated within the broader European discussion on research data stewardship, and the exploratory survey design is appropriate for mapping an emerging professional landscape. The paper also has important strengths in transparency: the authors make the underlying dataset, survey templates, and code available. The descriptive analysis is suitable for the size and nature of the sample, and the main message of the paper is useful: many institutions appear to have staff performing data-stewardship-related functions, but these roles are rarely formally recognised under that title.

 My recommendation is **Approved with Reservations**, because the main revisions I would request concern clarity, internal consistency, and the framing of some interpretations, rather than the overall validity of the study. First, the manuscript would benefit from a single, transparent accounting of denominators throughout the Results. Some changing sample sizes are understandable and appear to follow the survey logic, since later analyses are restricted to respondents reporting DS or equivalent roles and several figures explicitly use subset denominators. However, the reporting is not always easy to follow, and one inconsistency seems to require clarification: the paper states that 77 valid responses remained after cleaning; the institutional affiliation counts sum to 77 (33 from universities and 44 from public research institutions); yet the role counts reported in the same section (38 technical/administrative staff, 27 researchers, and 8 technologists) sum to 73. Because the methods state that roles were clustered into three macro-categories, the authors should clarify whether four responses were missing or unclassifiable, whether another category was omitted from the narrative, or whether the relevant denominator for that statement is smaller than 77. More generally, the manuscript would benefit from stating the applicable denominator more explicitly whenever a new subset of respondents is being analysed. Relatedly, there also appears to be some inconsistency in the description of the survey responses across sections: the Abstract mentions 88 responses, while the “Data cleaning and visualisation” section reports 84 collected responses before cleaning. Clarifying this progression of numbers (initial responses, cleaned dataset, and analysed subset) in a consistent way throughout the manuscript would improve transparency and readability.

 Second, the Zenodo citations should be corrected and made internally consistent. In the Methods, the survey templates and dataset are cited as *Caldoni et al. (2025a)*, but in the reference list *Caldoni et al. (2025a)* corresponds to the *Manifesto of the “Italian data steward community”*. The data and software availability section instead points to *Caldoni et al. (2025b)* for the data package and lists the survey templates there, but the DOI in that section does not match the DOI given in the reference list. This appears to be a citation and findability issue rather than a lack of data sharing, but it should be corrected so that readers can unambiguously locate the survey templates, dataset, and code.

 Third, the methods and limitations would benefit from a little more explanation in places. It would be helpful to explain why the survey was reopened, to clarify more explicitly how the activity list used in Figure 7 was compiled, and to discuss the likely effects of dissemination through Open Science-oriented mailing lists and related channels on the composition of the respondent pool. The paper already acknowledges that the sample covers only a limited number of institutions, and a slightly fuller discussion of representativeness and self-selection would strengthen the manuscript further.

 Finally, I encourage the authors to soften a few interpretive statements in the Discussion and Conclusions so that they remain closely aligned with what a descriptive, self-selected survey can support. In particular, some wording could be made more cautious where the manuscript infers broader institutional visibility or communication patterns, suggests a “growing emphasis” on the technical dimension, or describes the proposed activity list as exhaustive. These points do not undermine the usefulness of the study, but a more restrained phrasing would make the conclusions stronger and more precise. With these revisions, I think the article would make a useful contribution to the literature on data stewardship and research support in national contexts.

If applicable, is the statistical analysis and its interpretation appropriate?

Yes

Is the study design appropriate and is the work technically sound?

Yes

Is the work clearly and accurately presented and does it engage with the current literature?

Partly

Are the conclusions drawn adequately supported by the results?

Partly

Are sufficient details of methods and analysis provided to allow replication by others?

Yes

Are all the source data and materials underlying the results available?

Yes

Reviewer Expertise:

My research and professional activities focus on research data management, data stewardship, and data management planning. I contribute to the development of the Data Stewardship Wizard tool and participate in ELIXIR and national initiatives related to data stewardship and RDM support in research institutions.

I confirm that I have read this submission and believe that I have an appropriate level of expertise to confirm that it is of an acceptable scientific standard, however I have significant reservations, as outlined above.

# Response

**Published:** 2026-07-22

**Authors:** Giulia Caldoni

**Affiliations:** University of Bologna, Bologna, Emilia-Romagna, Italy

*We thank the referee for the encouraging feedback and for the thoughtful suggestions. They allowed us to improve the text significantly, and to clarify some aspects that deserved more space and attention.* *Below, we provide a detailed point-by-point response to each comment. Our responses follow each of the reviewer’s comments.* This article presents a valuable first national survey of data stewardship in Italian universities and public research institutions. The topic is timely and relevant, the manuscript is well situated within the broader European discussion on research data stewardship, and the exploratory survey design is appropriate for mapping an emerging professional landscape.

 The paper also has important strengths in transparency: the authors make the underlying dataset, survey templates, and code available. The descriptive analysis is suitable for the size and nature of the sample, and the main message of the paper is useful: many institutions appear to have staff performing data-stewardship-related functions, but these roles are rarely formally recognised under that title.

 My recommendation is Approved with Reservations, because the main revisions I would request concern clarity, internal consistency, and the framing of some interpretations, rather than the overall validity of the study. First, the manuscript would benefit from a single, transparent accounting of denominators throughout the Results. Some changing sample sizes are understandable and appear to follow the survey logic, since later analyses are restricted to respondents reporting DS or equivalent roles and several figures explicitly use subset denominators.

 However, the reporting is not always easy to follow, and one inconsistency seems to require clarification: the paper states that 77 valid responses remained after cleaning; the institutional affiliation counts sum to 77 (33 from universities and 44 from public research institutions); yet the role counts reported in the same section (38 technical/administrative staff, 27 researchers, and 8 technologists) sum to 73. Because the methods state that roles were clustered into three macro-categories, the authors should clarify whether four responses were missing or unclassifiable, whether another category was omitted from the narrative, or whether the relevant denominator for that statement is smaller than 77. More generally, the manuscript would benefit from stating the applicable denominator more explicitly whenever a new subset of respondents is being analysed. Relatedly, there also appears to be some inconsistency in the description of the survey responses across sections: the Abstract mentions 88 responses, while the “Data cleaning and visualisation” section reports 84 collected responses before cleaning. Clarifying this progression of numbers (initial responses, cleaned dataset, and analysed subset) in a consistent way throughout the manuscript would improve transparency and readability.

**Authors’ response:** We thank the reviewer for the very thorough comment, which helped us revise the consistency of the results that we considered to respond to each research question. Particularly, we identified and corrected some typos which hindered the understanding:

- Role sum: we reported 8 technologists while 12 was the correct number, hence 77 is the correct total. The typo error has now been corrected.
- Number of total responses: 88 is the correct number, now reported in the text.

We also specified, when answering each research question, the pool of responses we are basing our analysis on in order to improve clarity. Second, the Zenodo citations should be corrected and made internally consistent. In the Methods, the survey templates and dataset are cited as *Caldoni et al. (2025a)*, but in the reference list *Caldoni et al. (2025a)* corresponds to the *Manifesto of the “Italian data steward community”*. The data and software availability section instead points to *Caldoni et al. (2025b)* for the data package and lists the survey templates there, but the DOI in that section does not match the DOI given in the reference list. This appears to be a citation and findability issue rather than a lack of data sharing, but it should be corrected so that readers can unambiguously locate the survey templates, dataset, and code.

**Authors’ response:** We thank the reviewer for the observation. The references have been corrected in the revised version of the article. Third, the methods and limitations would benefit from a little more explanation in places. It would be helpful to explain why the survey was reopened, to clarify more explicitly how the activity list used in Figure 7 was compiled, and to discuss the likely effects of dissemination through Open Science-oriented mailing lists and related channels on the composition of the respondent pool. The paper already acknowledges that the sample covers only a limited number of institutions, and a slightly fuller discussion of representativeness and self-selection would strengthen the manuscript further.

**Authors’ response:** We thank the reviewer for the observation. To improve clarity, we revised the *Materials and methods* section to provide more details on the reasons for reopening the survey. We acknowledged and discussed potential limitations of the dissemination approach, including the survey being reopened, in the *Discussion* section. Finally, I encourage the authors to soften a few interpretive statements in the Discussion and Conclusions so that they remain closely aligned with what a descriptive, self-selected survey can support. In particular, some wording could be made more cautious where the manuscript infers broader institutional visibility or communication patterns, suggests a “growing emphasis” on the technical dimension, or describes the proposed activity list as exhaustive. These points do not undermine the usefulness of the study, but a more restrained phrasing would make the conclusions stronger and more precise. With these revisions, I think the article would make a useful contribution to the literature on data stewardship and research support in national contexts.

**Authors’ response:** We thank the reviewer for the suggestion. To improve the paper readability and the interpretation of the results, we carefully revised the manuscript to ensure the use of more cautious wording and to tone-down expressions that might have overemphasized the results.

# Reviewer report: Reviewer response for version 1

**DOI:** 10.21956/openreseurope.24014.r69757

**Published:** 2026-03-09

**Authors:** Angeliki Adamaki

**Affiliations:** 1 SciLifeLab Data Centre, Uppsala University (Ringgold ID: 463758), Uppsala, Sweden

**Summary**

 The article presents the results of a national survey exploring the presence, roles, and activities of professionals involved in research data management (RDM) in Italian universities and research institutions. The survey collected 77 valid responses (from 49 distinct institutions based on the CSV file and the ROR IDs provided) and provides an initial overview of how data stewardship-related tasks are currently distributed across institutional roles. The findings show that several institutions (25 based on the ROR IDs provided in the CSV data file and filtered for those knowing that they have DSs in their institutions) rely on staff performing data stewardship activities, such as support/guidance for FAIR data practices, data management plans, training, and policy guidance, although these responsibilities are not always formally recognised under the title of data steward. The study highlights a fragmented professional landscape and emphasises the need for clearer role definitions and professional pathways. At the same time, the work has potential impact beyond the Italian context, as it provides a landscape analysis at national level, contributes to community-building efforts through the creation of the Italian Data Steward Community, and proposes a methodological approach that could support similar mapping exercises in other countries.

**General Comments**

 The article has the potential to generate significant impact, as the survey could contribute to:

- landscape analyses of data stewardship practices following a reusable approach,
- community-building efforts,
- the development of a roadmap for the professionalisation of data stewardship.

Some statements in the article appear to reflect community experience rather than being directly supported by the presented data. Where possible, the authors should clarify this. Given the relatively small sample size (for statistical analysis), some statistical interpretations in the manuscript should be treated with caution. Qualitative expressions such as “a few” or “substantial” or “significant” can be difficult to interpret without corresponding numerical values (e.g. providing absolute numbers alongside percentages) which help readers better assess the significance of the results. Some recommendations for improvements are given below, considering the sample size and the visualisation options.

 Throughout the manuscript, the activities of data stewards are frequently described as providing “support” to researchers. While this term is common in RDM contexts, it may underestimate the broader role that data stewards often play (as also discussed in the article). In many cases, data stewards act not only as support staff but also as enablers of research, contributing to the development of infrastructures and relevant policies, and the design of research environments, frameworks and workflows, that make FAIR and sustainable data practices possible.

 The authors also mention “policy” in some parts of the text (e.g. “policy consultation”, “policymakers”), it will be good to clarify if they mean data policy, institutional policy, Open Science policy etc. GARR (pg. 5) is mentioned without explanation of the acronym and/or link.

 The article builds on the assumption that the importance of RDM is recognised within institutions (e.g. beginning of section “Data stewardship in Italy”, and in “Discussion”). It would be interesting to see (perhaps in a linked, follow up analysis) how this recognition emerged in the Italian context, and which factors contributed to it. For example:

- the role of research infrastructures (RIs),
- the influence of European policies or funding programmes,
- the identification of researchers’ needs perhaps via institutional initiatives.

Future developments could also consider connections with initiatives such as research assessment reform and recognition frameworks, which may influence how such roles are acknowledged within academic institutions.

**Questions (Q)**

 Q1: (related to respondents) Although it becomes clear as the analysis is presented, the authors can clarify early in the survey description that the participants were not necessarily data stewards themselves, and they replied either representing their institutions or based on their personal experiences.

 Q2: (related to EUSurvey) Are there technical or ethical considerations that others should know if they want to replicate the methodology?

 Q3: (related to Figure 1) Can the authors comment on survey coverage relative to the number of universities and research infrastructures in each region? This would help assess how representative the survey is. The figure helps the reader understand the sample, but not the data coverage. Of course each institution might have diverse departments with different needs in data stewardship, and only one response from a specific organisation might not be representative, so this should be clarified as well.

 Q4: Instead of excluding data from part of the analysis, would it be possible to present all responses, while clustering for individuals and institutional representatives? If subset of the dataset eventually is used (as it is now), the authors should explain this choice.

 Q5: (pg 7) “The majority of the respondents belong to.”, the numbers in parentheses add up to 73, but total number is 77?

 Q6: (Figures 2-3) Would the authors consider combining the figures in e.g. clustered or stacked bar chart? While pie charts are commonly used to visualize percentage, including counts alongside percentages will improve interpretability.

 Q7: (Figure 4a) When looking at the free-text responses there is at least one response where the participant writes that there is one officially identified DS and then other cases in their organisation, although they earlier answer “No” to whether there is a formally identified DS. Since the sample size is small, such details change the results presented. Besides this, the data shows that the respondents come from different institutions, which shows good data coverage and the authors could consider mentioning this.

 Q8: (General recommendation on figures) The authors may consider adapting the type of visualisation to the number of categories presented. For statistics with only a few categories (e.g., 2–4), pie charts can be effective, provided that absolute counts are shown alongside percentages. For analyses involving larger numbers of categories, horizontal bar charts may be more appropriate (absolute counts shown alongside percentages), as they allow category labels to be placed next to the bars, improving readability.

 Q9: (pg 8) The text describing Figure 6 can be improved if rephrased. The numbers in parentheses add up to 60, but these cannot be the respondents (n=38). The authors probably list the answers they collected as each respondent could give multiple answers.

 Q10: (Figure 5) Similar comments as in Q8 about the visualisation. If there are 13 respondents, then 1/13 is the minimum we expect. How did the 5% shown on the pie chart result?

 Q11: (Figure 7 and corresponding text) The authors mention percentages in the text, which are not shown on the Figure or in a table, and it’s not very easy to follow. On the figure it’s probably not necessary to mention the numbers at each bar, as they don’t add up to the number of respondents (multiple answers). These could be changed to match the text. If the aim of this part of the analysis is to show how the tasks of a DS are distributed, then the authors might consider describing this as e.g. RDM tasks are reported in x% of cases together with DMP, y% also involves training, etc.

 Q12: (Figure 8) Would it be interesting to distinguish between Unis and RIs?

 Q13: (Figure 9b) Similar comments as in Q8 regarding the visualisation in pie chart.

 Q14: (pg 12) “Conversely, it is notable that about 35%..”, although it’s indeed 7/20 responses, those do not correspond to 35% of the survey responses as the observation only refers to those representing their institutions. The authors might consider rephrasing this part, and perhaps also mentioning that these 20 responses seem to be from 20 different organisations.

 Q15: (pg 12) “..we can hypothesise..”, is this an assumption based on the authors’ experience from the community activities and fora?

 Q16: (pg 12) “..who answered in a personal capacity may reflect..”, could this be also because of weaknesses in the internal structure of each organisation, no mandate, etc.?

 Q17: (pg 12) “..difficulties in recognising who qualifies as a DS..”, can the authors elaborate?

 Q18: (pg 13) “..Additionally the notable representation of respondents..”, how is the role of the respondents relevant to the survey?

 Q19: (pg 13) There are some statements that are given as facts, while they could be better expressed as possible explanations based on the authors’ experience from their work, the DS community, etc. The survey presented here is a snapshot of time, and it would be interesting to see how these observations change and hopefully evolve.

If applicable, is the statistical analysis and its interpretation appropriate?

Partly

Is the study design appropriate and is the work technically sound?

Yes

Is the work clearly and accurately presented and does it engage with the current literature?

Yes

Are the conclusions drawn adequately supported by the results?

Partly

Are sufficient details of methods and analysis provided to allow replication by others?

Yes

Are all the source data and materials underlying the results available?

Yes

Reviewer Expertise:

Research Data Management and Open Science, with a focus on Research Infrastructures and community/service development.

I confirm that I have read this submission and believe that I have an appropriate level of expertise to confirm that it is of an acceptable scientific standard, however I have significant reservations, as outlined above.

# Response

**Published:** 2026-07-22

**Authors:** Giulia Caldoni

**Affiliations:** University of Bologna, Bologna, Emilia-Romagna, Italy

*We thank the referee for the encouraging feedback and for the thoughtful suggestions. They allowed us to improve the text significantly, and to clarify some aspects that deserved more space and attention.* *Below, we provide a detailed point-by-point response to each comment. Our responses follow each of the reviewer’s comments.*

**Summary** The article presents the results of a national survey exploring the presence, roles, and activities of professionals involved in research data management (RDM) in Italian universities and research institutions. The survey collected 77 valid responses (from 49 distinct institutions based on the CSV file and the ROR IDs provided) and provides an initial overview of how data stewardship-related tasks are currently distributed across institutional roles. The findings show that several institutions (25 based on the ROR IDs provided in the CSV data file and filtered for those knowing that they have DSs in their institutions) rely on staff performing data stewardship activities, such as support/guidance for FAIR data practices, data management plans, training, and policy guidance, although these responsibilities are not always formally recognised under the title of data steward.

 The study highlights a fragmented professional landscape and emphasises the need for clearer role definitions and professional pathways. At the same time, the work has potential impact beyond the Italian context, as it provides a landscape analysis at national level, contributes to community-building efforts through the creation of the Italian Data Steward Community, and proposes a methodological approach that could support similar mapping exercises in other countries.

**General Comments** The article has the potential to generate significant impact, as the survey could contribute to:

- landscape analyses of data stewardship practices following a reusable approach,
- community-building efforts,
- the development of a roadmap for the professionalisation of data stewardship.

Some statements in the article appear to reflect community experience rather than being directly supported by the presented data. Where possible, the authors should clarify this. Given the relatively small sample size (for statistical analysis), some statistical interpretations in the manuscript should be treated with caution. Qualitative expressions such as “a few” or “substantial” or “significant” can be difficult to interpret without corresponding numerical values (e.g. providing absolute numbers alongside percentages) which help readers better assess the significance of the results. Some recommendations for improvements are given below, considering the sample size and the visualisation options.

**Authors’ response: ** We thank the reviewer for the suggestions. Accordingly, we have revised the wording throughout the manuscript to avoid any implication of inferential statistical significance and to more accurately reflect the descriptive and exploratory nature of the study. We have deleted qualitative expressions where appropriate and consistently reported absolute numbers alongside percentages throughout the manuscript. We also acknowledge the reviewer's concern regarding the relatively small sample size and have more explicitly addressed this limitation in the *Discussion *section.   Throughout the manuscript, the activities of data stewards are frequently described as providing “support” to researchers. While this term is common in RDM contexts, it may underestimate the broader role that data stewards often play (as also discussed in the article). In many cases, data stewards act not only as support staff but also as enablers of research, contributing to the development of infrastructures and relevant policies, and the design of research environments, frameworks and workflows, that make FAIR and sustainable data practices possible. The authors also mention “policy” in some parts of the text (e.g. “policy consultation”, “policymakers”), it will be good to clarify if they mean data policy, institutional policy, Open Science policy etc. GARR (pg. 5) is mentioned without explanation of the acronym and/or link.

**Authors’ response:** We thank the reviewer for the observation, which helped us improve the precision of the manuscript. To better reflect the scope and specificity of the profession, we replaced several occurrences of “support to data management” with the terms “data stewardship” and “DS role(s)” throughout the text. Moreover, we clarified the meaning of “policies” in the *Introduction* section. explained acronyms. We revised the text to explain acronyms, except “GARR” which can’t be explained as it is not an acronym. We provided a link to the Consortium GARR webpage for clarity. The article builds on the assumption that the importance of RDM is recognised within institutions (e.g. beginning of section “Data stewardship in Italy”, and in “Discussion”). It would be interesting to see (perhaps in a linked, follow up analysis) how this recognition emerged in the Italian context, and which factors contributed to it. For example:

- the role of research infrastructures (RIs),
- the influence of European policies or funding programmes,
- the identification of researchers’ needs perhaps via institutional initiatives.

Future developments could also consider connections with initiatives such as research assessment reform and recognition frameworks, which may influence how such roles are acknowledged within academic institutions.

**Authors’ response**: We thank the reviewer for this suggestion. Although we decided to remove from the manuscript the reference to a future, expanded version of the survey, this remains one of the objectives of the Italian Data Stewards Community, whose establishment was facilitated by this initial survey. We will take the reviewer’s suggestions into account when planning future survey activities.

**Questions (Q)** Q1: (related to respondents) Although it becomes clear as the analysis is presented, the authors can clarify early in the survey description that the participants were not necessarily data stewards themselves, and they replied either representing their institutions or based on their personal experiences.

**Authors’ response: ** We thank the reviewer for the observation. We clarified this aspect in the *Materials and Methods* section when describing the structure of the questionnaire.    

 Q2: (related to EUSurvey) Are there technical or ethical considerations that others should know if they want to replicate the methodology?

**Authors’ response: ** We thank the reviewer for the question. EUSurvey is an open-source platform provided by the European Commission, which ensures compliance with European data protection standards. It also allows anonymous responses, with no personal or sensitive data being collected or requested. This has been added in the ethical statement of the article.

 Q3: (related to Figure 1) Can the authors comment on survey coverage relative to the number of universities and research infrastructures in each region? This would help assess how representative the survey is. The figure helps the reader understand the sample, but not the data coverage. Of course each institution might have diverse departments with different needs in data stewardship, and only one response from a specific organisation might not be representative, so this should be clarified as well.

**Authors’ response:** We thank the reviewer for this interesting observation. We agree that assessing the geographical coverage of the survey at the regional level would provide additional insight, particularly considering that responses were obtained from at least one university or research organisation in all but three Italian regions. However, conducting such an analysis would require a more detailed assessment of institutional coverage, as the Italian research landscape is highly fragmented and organisationally complex. For example, the National Research Council of Italy (CNR), which counts as a single research organisation, comprises more than 200 seats distributed across the country. To address the reviewer(s) question, we have revised the manuscript to specify that responses were obtained from 17 of the 104 universities and research-performing organisations operating in Italy. We have discussed that this underrepresentation may affect the generalisation of the findings. We also clarified that the primary aim of the study is to provide a snapshot of current practices rather than a fully comprehensive or statistically representative national mapping. These additions can be found in the *Discussion *section.

 Q4: Instead of excluding data from part of the analysis, would it be possible to present all responses, while clustering for individuals and institutional representatives? If subset of the dataset eventually is used (as it is now), the authors should explain this choice.

**Authors’ response: ** We thank the reviewer for the suggestion. We deeply revised the Analysis of Survey Results section to clearly specify which subset of the dataset was used to answer every Research Question (RQ). We decided to consider only responses provided on behalf of organisations (n=20) to answer RQ1 (“To what extent are DSs formally integrated in research performing organizations in Italy?”) as our interest was focused on capturing the formal and institutional definition of the DS role. Further RQs aimed, on the other hand, to characterize the professional identity of DSs, hence we based our analysis on the total of answers received.

 Q5: (pg 7) “The majority of the respondents belong to.”, the numbers in parentheses add up to 73, but total number is 77?

**Authors’ response: ** We thank the reviewer for the comment. There was a typo concerning the number of Technologists: 8 instead of 12. The typo error has now been corrected.

 Q6: (Figures 2-3) Would the authors consider combining the figures in e.g. clustered or stacked bar chart? While pie charts are commonly used to visualize percentage, including counts alongside percentages will improve interpretability.

**Authors’ response: ** We thank the reviewer for the observation, shared also by referee 2. We removed figure 2 and reported the numbers only in the text. We maintained figure 3 as we think it’s relevant to give a visual snapshot of the number of formally recognized data stewards in the national landscape. We also removed pie charts and used tables instead.

 Q7: (Figure 4a) When looking at the free-text responses there is at least one response where the participant writes that there is one officially identified DS and then other cases in their organisation, although they earlier answer “No” to whether there is a formally identified DS. Since the sample size is small, such details change the results presented. Besides this, the data shows that the respondents come from different institutions, which shows good data coverage and the authors could consider mentioning this.

**Authors’ response: ** We thank the reviewer for the very attentive comment. When answering RQ1, we based our quantitative analyses on responses to the structured survey questions. In the specific case that is pointed out, the respondent selected “No” when asked whether the individuals performing data stewardship functions were formally identified as data stewards. Furthermore, the accompanying comment suggests that, although at least one individual may hold the title of Data Steward, data stewardship activities within the organisation are predominantly carried out by personnel who are not formally designated as such. Therefore, the response was considered consistent with the intended interpretation of the survey question and was classified in the “No” category. According to the reviewer’s suggestion, we commented on the good institutional data coverage at the beginning of the *Analysis of Survey Results* section.

 Q8: (General recommendation on figures) The authors may consider adapting the type of visualisation to the number of categories presented. For statistics with only a few categories (e.g., 2–4), pie charts can be effective, provided that absolute counts are shown alongside percentages. For analyses involving larger numbers of categories, horizontal bar charts may be more appropriate (absolute counts shown alongside percentages), as they allow category labels to be placed next to the bars, improving readability.

**Authors’ response: ** We thank the reviewer for this comment. Figure 4b has been converted into a table to clearly present the different categories and the number of responses for each.

 Q9: (pg 8) The text describing Figure 6 can be improved if rephrased. The numbers in parentheses add up to 60, but these cannot be the respondents (n=38). The authors probably list the answers they collected as each respondent could give multiple answers.

**Authors’ response: ** We thank the reviewer for the comment. For the analysis now reported in RQ3 and RQ4 we considered all respondents who reported the presence of Data Stewards (or equivalent roles) within their organisation (n = 38), regardless of whether the questionnaire had been completed on behalf of the institution or in an individual capacity. Multiple (non–mutually exclusive) responses were allowed. To avoid misunderstanding, we have clarified this selection criterion in the manuscript and in the corresponding figure captions.

 Q10: (Figure 5) Similar comments as in Q8 about the visualisation. If there are 13 respondents, then 1/13 is the minimum we expect. How did the 5% shown on the pie chart result?

**Authors’ response: ** We thank the reviewer for the comment. Figure 5 has been converted into a table to better evaluate the number of responses for each role. Multiple (non-mutually exclusive) responses were allowed.

 Q11: (Figure 7 and corresponding text) The authors mention percentages in the text, which are not shown on the Figure or in a table, and it’s not very easy to follow. On the figure it’s probably not necessary to mention the numbers at each bar, as they don’t add up to the number of respondents (multiple answers). These could be changed to match the text. If the aim of this part of the analysis is to show how the tasks of a DS are distributed, then the authors might consider describing this as e.g. RDM tasks are reported in x% of cases together with DMP, y% also involves training, etc.

**Authors’ response:** We thank the reviewer for the suggestion. Accordingly, we have reported absolute numbers alongside percentages throughout the manuscript.

 Q12: (Figure 8) Would it be interesting to distinguish between Unis and RIs?

**Authors’ response: ** We thank the reviewer for the suggestion. We did not report results stratified by institution type, as this distinction was not central to the study objectives and did not contribute substantially to the overall interpretation of data stewardship distribution in Italy.

 Q13: (Figure 9b) Similar comments as in Q8 regarding the visualisation in pie chart.

**Authors’ response: ** We thank the reviewer for the comment. Figure 9b has been converted into a table to better present results as absolute numbers. Multiple (non-mutually exclusive) responses were allowed.

 Q14: (pg 12) “Conversely, it is notable that about 35%..”, although it’s indeed 7/20 responses, those do not correspond to 35% of the survey responses as the observation only refers to those representing their institutions. The authors might consider rephrasing this part, and perhaps also mentioning that these 20 responses seem to be from 20 different organisations. **Authors’ response: ** We thank the reviewer for this comment. The sentence has been rephrased as follows: “ *We also note that 35% of the respondents (7 out of 20, Figure 3) reported the absence of DSs in their institution, suggesting uneven levels of awareness on this topic.*” We did not further comment on the responding organisations.

 Q15: (pg 12) “..we can hypothesise..”, is this an assumption based on the authors’ experience from the community activities and fora?

**Authors’ response**: We thank the reviewer for the observation. We carefully revised the text to avoid over-interpretations of the results, hence the specific sentence mentioned has been deleted.

 Q16: (pg 12) “..who answered in a personal capacity may reflect..”, could this be also because of weaknesses in the internal structure of each organisation, no mandate, etc.?

**Authors’ response:** We have included the reviewer's suggestion in the updated version of the article. Now the sentence has been rephrased as follows: “ *We observed that only a minority of respondents participated in the survey on behalf of their institution. Consistent with the European trends reporting a correlation between institutional maturity in RDM and the adoption of DS roles (Gruber, 2021; Jetten et al., 2021; Seidlmayer et al., 2023; Wildgaard et al., 2020), it is reasonable to infer that institutions with higher levels of awareness and engagement in RDM are more likely to be developing or implementing structured RDM support services and to have designated a representative for survey completion.*”

 Q17: (pg 12) “..difficulties in recognising who qualifies as a DS..”, can the authors elaborate? **Authors’ response:** We thank the reviewer for this comment. The sentence has been removed, and the key concepts have been revised and rephrased throughout the manuscript.

 Q18: (pg 13) “..Additionally the notable representation of respondents..”, how is the role of the respondents relevant to the survey?

**Authors’ response: ** We thank the reviewer for the comment. Respondents' roles were collected to contextualize the survey findings. Since participation was open to professionals beyond formal data stewards, the results reflect a broader data stewardship community. The survey did not explicitly assess whether responses were based on personal experience. We modified the text to address this point both in the *Analysis of Survey Results* and in the *Discussion* sections.

 Q19: (pg 13) There are some statements that are given as facts, while they could be better expressed as possible explanations based on the authors’ experience from their work, the DS community, etc. The survey presented here is a snapshot of time, and it would be interesting to see how these observations change and hopefully evolve.

**Authors’ response: ** We thank the reviewer for this comment. We revised the manuscript and rephrased some sentences to avoid over-interpretation of the results. Although we decided to remove from the manuscript the reference to a future, expanded version of the survey, this remains one of the objectives of the Italian Data Stewards Community, whose establishment was facilitated by this initial survey. We will take the reviewer’s suggestions into account when planning future survey activities.

# Reviewer report: Reviewer response for version 1

**DOI:** 10.21956/openreseurope.24014.r69765

**Published:** 2026-03-09

**Authors:** Louise Saul

**Affiliations:** 1 University of Southampton, Southampton, England, UK

This article describes a survey that was circulated to research institutions in Italy, via a multifaceted snowball approach, within 2 two-month time periods in the year 2023.

 It builds upon previous work by other groups to define and professionalise the role of Data Steward in the research setting, particularly looking at work in the Netherlands (1), Germany (2), and Denmark (3) and seeks to undertake the same project in an Italian setting. The introduction of this manuscript frames the narrative and need for this work.

 This is an extremely timely piece of work as various EU projects came to a close there was a realisation of the requirement for a continued effort to define the Data Steward role across Europe. However, whilst there needs to be a united effort to ensure that a ubiquitous language is used to describe this role, each nation's research ecosystem will be subtly unique. Understanding this variability is key to ensuring a cohesive approach to embedding Data Stewardship, which necessitates an understanding of the current landscape.

 This work seeks to describe the current status of both Data Stewards and people who undertake work that would be completed by Data Stewards at research institutions in Italy, capturing information about their activities, responsibilities, geographical location, official roles, training requirements, recognition for their work, and area in which they are located.

 This is a very important piece of work; the survey is comprehensive without being a burden for the user to complete. I was particularly interested in the approach taken to include a description of a 'Data Steward' at the beginning of the survey; it may have been interesting to allow respondent to comment upon this definition, however I appreciate that may complicate the analysis. It would be interesting to understand the rationale for developing the survey and inclusion of questions, however in emerging fields there is often not sufficient information to do so and as stated above, often national landscapes can be unique enough to prevent a direct transposition. I assume that the survey design was based upon previous work (4). 

 Promoting reproducibility and data re-use for systematic review and meta-analyses, the authors have made the data, survey, and analysis code fully available with persistent identifiers, also including information about the methods used to clean the data. It would have been interesting to include more information about the institution 'types' that respondent worked at (i.e. Independent research institute, higher education, government sponsored institute) in order to fully understand the context in which the answers were given.

 The way the responses have been summarised in this manuscript presents an interesting and useful picture that will be useful to compare to other countries' summaries of the same role. I have some questions over the breakdown of responses for figure 4b and figure 5, where descriptive statistic may not be helpful for so few respondents and a table or similar may be more appropriate. I am also unclear as to why only 38 respondents (I presume identified from the 'Technical/Administrative staff' cluster) were included in the analysis for figure 6/7/8/9, but this may be as a result of my own understanding. The results were illuminating and are very valuable for understanding the role of Data Steward in Italian research organisations.

 I found the discussion to be very insightful and appreciated the summary that the authors provided. I look forward to seeing outputs from the Italian Data Stewards Community.

If applicable, is the statistical analysis and its interpretation appropriate?

Partly

Is the study design appropriate and is the work technically sound?

Yes

Is the work clearly and accurately presented and does it engage with the current literature?

Yes

Are the conclusions drawn adequately supported by the results?

Yes

Are sufficient details of methods and analysis provided to allow replication by others?

Yes

Are all the source data and materials underlying the results available?

Yes

Reviewer Expertise:

Data Stewardship skills and careers

I confirm that I have read this submission and believe that I have an appropriate level of expertise to confirm that it is of an acceptable scientific standard.

## References

[1] : Professionalising data stewardship in the Netherlands. Competences, training and education. Dutch roadmap towards national implementation of FAIR data stewardship (1.1). *https://doi.org/10.5281/zenodo.4623713* .2021;

[2] : RDA Professionalising Data Stewardship IG - What does a career track for data stewards look like?. *https://doi.org/10.15497/RDA00102* .2024;

[3] : National Coordination of Data Steward Education in Denmark: Final report to the National Forum for Research Data Management (DM Forum) (Version 1). *https://doi.org/10.5281/zenodo.3609516* .2020;

[4] : Forschung unterstützen - Empfehlungen für Data Stewardship an akademischen Forschungsinstitutionen. *https://doi.org/10.4126/FRL01-006441397* .2023;

# Response

**Published:** 2026-07-22

**Authors:** Giulia Caldoni

**Affiliations:** University of Bologna, Bologna, Emilia-Romagna, Italy

We sincerely thank the reviewer for the positive feedback and insightful comments, which enabled us to refine the manuscript. Below, we provide a detailed point-by-point response to each comment. Our responses follow each of the reviewer’s comments.    

 This article describes a survey that was circulated to research institutions in Italy, via a multifaceted snowball approach, within 2 two-month time periods in the year 2023. It builds upon previous work by other groups to define and professionalise the role of Data Steward in the research setting, particularly looking at work in the Netherlands (1), Germany (2), and Denmark (3) and seeks to undertake the same project in an Italian setting. The introduction of this manuscript frames the narrative and need for this work. This is an extremely timely piece of work as various EU projects came to a close there was a realisation of the requirement for a continued effort to define the Data Steward role across Europe.

 However, whilst there needs to be a united effort to ensure that a ubiquitous language is used to describe this role, each nation's research ecosystem will be subtly unique. Understanding this variability is key to ensuring a cohesive approach to embedding Data Stewardship, which necessitates an understanding of the current landscape. This work seeks to describe the current status of both Data Stewards and people who undertake work that would be completed by Data Stewards at research institutions in Italy, capturing information about their activities, responsibilities, geographical location, official roles, training requirements, recognition for their work, and area in which they are located.

 This is a very important piece of work; the survey is comprehensive without being a burden for the user to complete. I was particularly interested in the approach taken to include a description of a 'Data Steward' at the beginning of the survey; it may have been interesting to allow respondent to comment upon this definition, however I appreciate that may complicate the analysis. It would be interesting to understand the rationale for developing the survey and inclusion of questions, however in emerging fields there is often not sufficient information to do so and as stated above, often national landscapes can be unique enough to prevent a direct transposition. I assume that the survey design was based upon previous work (4).

**Authors’ response:** We thank the reviewer for the observation. We made the rationale more explicit in the *Materials and Methods* section. The authors’ direct professional experience was complemented by insights drawn from international reports, allowing to transfer international experiences to the Italian context. Promoting reproducibility and data re-use for systematic review and meta-analyses, the authors have made the data, survey, and analysis code fully available with persistent identifiers, also including information about the methods used to clean the data. It would have been interesting to include more information about the institution 'types' that respondent worked at (i.e. Independent research institute, higher education, government sponsored institute) in order to fully understand the context in which the answers were given. The way the responses have been summarised in this manuscript presents an interesting and useful picture that will be useful to compare to other countries' summaries of the same role. I have some questions over the breakdown of responses for figure 4b and figure 5, where descriptive statistic may not be helpful for so few respondents and a table or similar may be more appropriate.

**Authors’ response:** We thank the reviewer for the observation. We decided to use tables to report the breakdown data that were previously presented in the pie charts of figures 4b and 5b. I am also unclear as to why only 38 respondents (I presume identified from the 'Technical/Administrative staff' cluster) were included in the analysis for figure 6/7/8/9, but this may be as a result of my own understanding. The results were illuminating and are very valuable for understanding the role of Data Steward in Italian research organisations.

**Authors’ response:** We thank the reviewer for the observation, which helped us clarify the reporting of results. For the analysis now reported in RQ3 and RQ4 we considered all respondents who reported the presence of Data Stewards (or equivalent roles) within their organisation (n = 38), regardless of whether the questionnaire had been completed on behalf of the institution or in an individual capacity. Multiple (non–mutually exclusive) responses were allowed. To avoid misunderstanding, we have clarified this selection criterion in the manuscript and in the corresponding figure captions. I found the discussion to be very insightful and appreciated the summary that the authors provided. I look forward to seeing outputs from the Italian Data Stewards Community.

**Authors’ response:** We thank the reviewer for the positive assessment of the value of these results. We are happy to share that all the outputs from the Italian Data Steward Community are available in our Zenodo community: https://zenodo.org/communities/italiads/records?q=&l=list&p=1&s=10&sort=newest

# Reviewer report: Reviewer response for version 1

**DOI:** 10.21956/openreseurope.24014.r69761

**Published:** 2026-03-09

**Authors:** Antti M. Rousi

**Affiliations:** 1 Aalto University, Espoo, Espoo, Finland

Congratulations to the authors for this interesting research. This work was a pleasure to review. I agree that the present study -- along with its valuable open data -- could serve as a benchmark for similar national questionnaires mapping the state of data stewardship in other countries. Although the number responses may seem small, these respondents represent a highly specialized and rare group of professionals. I believe that the final version of this work could be indexed in ORE.

 However, I also believe this work must be improved before it can be accepted for indexing. Although my general sentiment for this research is positive and I wish to support the authors’ research, I recommend a major revision followed by a new round of reviews. I hope the authors find my suggestions helpful.

**Work title**

 The current manuscript is titled “Mapping data stewardship in Italy: Findings from the first national survey”. I understand the use of term “mapping” within this context, but I wonder if the title would work better with search engines without it. Also, the term “first” seems unnecessary in the title. I invite the authors to contemplate whether the title could be shortened into “Data stewardship in Italy: Findings from a national survey”.

**Terminology and RQs**

 The understandability of the article suffers from terminological discrepancies.

- The abstract states the research aim as to “map the presence, roles, and activities of data stewards or equivalent professionals across universities and research institutions, to provide an initial and systematic overview of the national landscape” 
- The introduction, however, seems to be built upon the three following core concepts of data stewardship, data stewardship profiles, and data stewardship service models
- The end of the Introduction states that “[…] the idea emerged to launch a public survey to map the state of data stewardship in Italian universities and research-performing institutions. A further objective of the survey was to assess the respondents’ willingness to join a national community of practice on RDM.”
- Furthermore, within the methodology section, the structure of the survey questionnaire is based on “Respondent Profiles” and “Institutional Characterisation”.

This terminological looseness is exacerbated by the fact that the research aim and research questions are not addressed in the article text.

 Looking at the survey questionnaire, the research aim and questions could perhaps be formulated, for instance, as follows: “The research aim of the present work is to investigate how data stewardship services are organised within Italian universities and research institutions. The research questions are posed as follows.

- RQ1 What kinds of professionals undertake data stewardship in Italian universities and research institutions?
- RQ2 Which service organizations employ the personnel undertaking data stewardship activities?
- RQ3 Which kinds of data stewardship support actions are these professionals currently undertaking?”

I believe the authors must:

- Provide a general introduction to their research aim and define their core terminology in the Introduction before the literature review segment
- Link the literature review with the core terminology defined in the Introduction (e.g. “The question of what kinds of professionals undertake data stewardship is addressed in the research branch focussing on DS profiles”; note! I am not sure if the latter is factually correct; it is more of a style example)
- Define their research aim and research questions in detail after the literature review segment (see the example RQs above)
- Make sure the methods section is terminologically aligned with the research aim and RQs
- Base the reporting of their survey results around the RQs, e.g., one Findings sub segment per RQ
- Briefly summarize the most important findings per RQ in the Discussion segment

**Literature review**

 The authors must improve their literature review. The draft only cites 17 works, which is a small amount for a research article. Furthermore, the draft mostly cites EOSC, RDA and national working group reports when of course the literature on data stewardship is more broad. A simple Google Scholar search with the term “data stewardship” produces several relevant works currently missing from cited references. Besides research articles focussing on data stewardship, also literature on how academic libraries have arranged RDM services are in my view relevant to this work. As a rule of thumb, the authors should strive to double the amount of cited references when adding research articles into the list of references.     

**Misc. notes and improvement suggestions**

- For a better flow of the current Introduction text, I suggest the authors experiment with removal of the subtitles (e.g. “International efforts to professionalise research data stewardship”). Furthermore, the authors could experiment with moving the current 2nd paragraph of introduction as the new 4th paragraph of introduction. The latter solution could improve the text flow introducing the concepts of DS profiles and DS service models.  
- I don’t believe one needs figures to illustrate yes/no questions or other survey questions with only two answer options. Figures 2 and 3 could thus be removed and this information presented in article text only  
- I would avoid pie charts in research articles (see e.g. https://zenodo.org/records/17550233). I suggest you would use column charts instead throughout the paper
- I suggest you state the limitations of your study as the second last paragraph of your discussion section, followed by a concluding paragraph. It is important to acknowledge the limitations of your work, but stating them at the beginning of the discussion can divert the attention from your main results
- After thorough revision of the article, I recommend sending it for professional English language editing

If applicable, is the statistical analysis and its interpretation appropriate?

Not applicable

Is the study design appropriate and is the work technically sound?

Yes

Is the work clearly and accurately presented and does it engage with the current literature?

Partly

Are the conclusions drawn adequately supported by the results?

Partly

Are sufficient details of methods and analysis provided to allow replication by others?

Yes

Are all the source data and materials underlying the results available?

Yes

Reviewer Expertise:

Data stewardship, Information science, scientific communication

I confirm that I have read this submission and believe that I have an appropriate level of expertise to confirm that it is of an acceptable scientific standard, however I have significant reservations, as outlined above.

# Response

**Published:** 2026-07-22

**Authors:** Giulia Caldoni

**Affiliations:** University of Bologna, Bologna, Emilia-Romagna, Italy

*We thank the referee for the encouraging feedback and for the thoughtful suggestions. They allowed us to improve the text significantly, and to clarify some aspects that deserved more space and attention.* *Below, we provide a detailed point-by-point response to each comment. Our responses follow each of the reviewer’s comments.*

**Work title** The current manuscript is titled “Mapping data stewardship in Italy: Findings from the first national survey”. I understand the use of term “mapping” within this context, but I wonder if the title would work better with search engines without it. Also, the term “first” seems unnecessary in the title. I invite the authors to contemplate whether the title could be shortened into “Data stewardship in Italy: Findings from a national survey”.

**Authors’ response: ** We thank the reviewer for the observation. We agree that the shorter version of the title is more appropriate, hence we accepted the suggested form “Data stewardship in Italy: Findings from a national survey”.

**Terminology and RQs**  The understandability of the article suffers from terminological discrepancies.

- The abstract states the research aim as to “map the presence, roles, and activities of data stewards or equivalent professionals across universities and research institutions, to provide an initial and systematic overview of the national landscape” 
- The introduction, however, seems to be built upon the three following core concepts of data stewardship, data stewardship profiles, and data stewardship service models
- The end of the Introduction states that “[…] the idea emerged to launch a public survey to map the state of data stewardship in Italian universities and research-performing institutions. A further objective of the survey was to assess the respondents’ willingness to join a national community of practice on RDM.”
- Furthermore, within the methodology section, the structure of the survey questionnaire is based on “Respondent Profiles” and “Institutional Characterisation”.

**Authors’ response: ** We thank the reviewer for their observations. We agree that the manuscript contained some terminological discrepancies that could affect its readability. To address this, we revised the text to consistently distinguish between data steward role(s) and data stewardship services/service models throughout the manuscript. We also revised the Introduction to improve conceptual coherence and ensure consistency across the Abstract, Introduction, and Methodology sections. This terminological looseness is exacerbated by the fact that the research aim and research questions are not addressed in the article text. Looking at the survey questionnaire, the research aim and questions could perhaps be formulated, for instance, as follows: “The research aim of the present work is to investigate how data stewardship services are organised within Italian universities and research institutions. The research questions are posed as follows.

- RQ1 What kinds of professionals undertake data stewardship in Italian universities and research institutions?
- RQ2 Which service organizations employ the personnel undertaking data stewardship activities?
- RQ3 Which kinds of data stewardship support actions are these professionals currently undertaking?”

**Authors’ response: ** We thank the reviewer for the suggestion. We made RQs explicit in the *Introduction* section. I believe the authors must:

- Provide a general introduction to their research aim and define their core terminology in the Introduction before the literature review segment
- Link the literature review with the core terminology defined in the Introduction (e.g. “The question of what kinds of professionals undertake data stewardship is addressed in the research branch focussing on DS profiles”; note! I am not sure if the latter is factually correct; it is more of a style example)
- Define their research aim and research questions in detail after the literature review segment (see the example RQs above)
- Make sure the methods section is terminologically aligned with the research aim and RQs
- Base the reporting of their survey results around the RQs, e.g., one Findings sub segment per RQ
- Briefly summarize the most important findings per RQ in the Discussion segment

**Authors’ response: ** We thank the reviewer for their observations. We have revised the *Analysis of Survey Results* section to organise more clearly the presentation of the findings around the four RQs, ensuring that each RQ is clearly addressed in a dedicated subsection. Furthermore, we have revised the *Discussion* section to summarise more explicitly the key findings in relation to each RQ.

**Literature review** The authors must improve their literature review. The draft only cites 17 works, which is a small amount for a research article. Furthermore, the draft mostly cites EOSC, RDA and national working group reports when of course the literature on data stewardship is more broad. A simple Google Scholar search with the term “data stewardship” produces several relevant works currently missing from cited references. Besides research articles focussing on data stewardship, also literature on how academic libraries have arranged RDM services are in my view relevant to this work. As a rule of thumb, the authors should strive to double the amount of cited references when adding research articles into the list of references.     

**Authors’ response: ** We thank the reviewer for the suggestion. The literature review has been revised accordingly, and 10 more references were added. Misc. notes and improvement suggestions

- For a better flow of the current Introduction text, I suggest the authors experiment with removal of the subtitles (e.g. “International efforts to professionalise research data stewardship”). Furthermore, the authors could experiment with moving the current 2nd paragraph of introduction as the new 4th paragraph of introduction. The latter solution could improve the text flow introducing the concepts of DS profiles and DS service models.

**Authors’ response: ** We thank the reviewer for the observation. We changed the order of the paragraphs as suggested to improve text flow. We decided to maintain the subtitles to identify paragraphs, as we believe it helps readability. 

- I don’t believe one needs figures to illustrate yes/no questions or other survey questions with only two answer options. Figures 2 and 3 could thus be removed and this information presented in article text only  
- I would avoid pie charts in research articles (see e.g. https://zenodo.org/records/17550233). I suggest you would use column charts instead throughout the paper

**Authors’ response: ** We thank the reviewer for the observation, shared also by referee 4. We removed figure 2 as suggested, while we maintained figure 3 as we think it’s relevant to give a visual snapshot of the number of formally recognized data stewards in the national landscape. We also removed pie charts and used tables instead.

- I suggest you state the limitations of your study as the second last paragraph of your discussion section, followed by a concluding paragraph. It is important to acknowledge the limitations of your work, but stating them at the beginning of the discussion can divert the attention from your main results

**Authors’ response: ** We thank the reviewer for the suggestion. We revised the *Discussion* section and reported the limitations of the study at the end of the section.

- After thorough revision of the article, I recommend sending it for professional English language editing

**Authors’ response: ** We thank the reviewer for the suggestion. The manuscript went through copyediting to enhance clarity and conciseness.

# Reviewer report: Reviewer response for version 1

**DOI:** 10.21956/openreseurope.24014.r69766

**Published:** 2026-03-03

**Authors:** Aoife Coffey, Pádraig Mac Aodhgáin

**Affiliations:** 1 University College Cork Library, University College Cork National University of Ireland University College Cork Boole Library (Ringgold ID: 494323), Cork, County Cork, Ireland

This manuscript presents the findings of the first national survey on data stewardship in Italian universities and research institutions. The survey responds to the growing need for research data management support in line with Open Research and the FAIR Principles.  The key findings suggest that data stewardship in Italy is not formalised, there is a spectrum of job titles and responsibilities and the backgrounds of those in data stewardship roles varies widely.  It concludes that there is a growing presence of data stewardship in Italy but that limited formal recognition of the role are preventing clear career pathways and recognition. Highlighting the need for national coordination to strengthen professional identities and recognition and work towards standardize practices.

**Is the work clearly and accurately presented, and does it engage with the current literature?**

 The work is well-grounded in relevant literature and contextualised within the European research landscape. We encourage the authors revisit the statement *“only 33% of the European respondents actually had the job title “data steward” *The figure mentioned does not seem to be supported by the data underlying the cited report and potentially this needs to be rephrased for accuracy. Data available here https://zenodo.org/records/10117910

**Is the study design appropriate and is the work technically sound?**

 The design is appropriate and socialised in relevant and available channels.

- The survey was reopened for a period could the reason and justification for this be included in the methods.
- Some additional details of the limitations of the survey would be useful such as an acknowledgement of the limitation of using mailing list which are open science focused so could be seen as self-selecting.
- We also suggest including details of how well the 77 responses represent the full Italian research ecosystem – number of eligible institutions vs the number of responses and an estimated national research population.
- Could the authors also clarify how the list of data stewardship activities outline in Figure 7 was complied.

**Are sufficient details of methods and analysis provided to allow replication by others?**

 The methodology is described in detail and is sound and appropriate.

 However, there could be some improvements.

- In figure 1 two of the data points beside the number 17 are the same for consistency should 2 be 1.5 times the size of 1. Pie charts for cities with 2-5 respondents were visualised as 1.5 the size of the units.
- Names of major cities could be included in figure 1 for those who are not familiar with the geography of Italy.
- Figure 8 – could the axis be explained perhaps include a legend to clarify what each point on the bar chart means.  

**Are all the source data and materials underlying the results available?**

 Yes all data and material have been shared

- The underlying data provides the survey questions in English but could the column headers in the dataset also be translated to English.

**If applicable, is the statistical analysis and its interpretation appropriate?**

 The analysis is descriptive which is appropriate given the profile of the data and  sample size.

- It may be helpful to refine some of the wording used in the descriptions of the findings. Softening the phrasing will ensure that the interpretations come across as balanced and precise. An example would be the phrase ‘striking majority’  which seems a bit of an overstatement.

**Are the conclusions drawn adequately supported by the results?**

 The authors acknowledge the limitations of the study in the conclusion, however there are several instances where the discussion appears to over-interpret or extend the findings beyond what can be supported by the data.  

- The conclusions section would benefit from being more concise and from reframing the content to highlight a small number of clear, well-defined take-home messages.  
- The conclusion section would also benefit from clarification of the authors definition of DS-like staff, DS- activities, DS-like responsibilities, DS-like professionals as they are mentioned in the discussion but not elsewhere. It would be good to under the meaning  of these phases in the context of the survey.
- *“In the future, we plan to perform another survey including as many universities and research institutions as possible.” *We suggest removing this as it is should be an aim from the outset.
- We suggest that this statement is revisited *“On the other hand, the large proportion of respondents who answered in a personal capacity may reflect limited institutional awareness/policies related to RDM needs or a lack of internal communication about existing practices.” * It is an over interpretation of the findings but could be rephrased as question.
- For the following line it is not clear how this assertion  is supported by figure 6 and 7.  “ *According to the responses that we gathered here, a DS is a professionals with (at least one or more) cross-cutting skills in RDM (disciplinary, IT and technical, legal) and who very often acts as a bridge between researchers (i.e. producers and users of research data), infrastructures and research organisations (Figure 6 & Figure 7).”*
- The suggestion that the list of data stewardship task was *‘exhaustive’* seems to be an overreach considering the sample size and the early stage of data stewardship socialization and adoption in Italy in 2023 as described by the author themselves. Also in light of earlier statement such as *“* *DS-like activities are performed informally or distributed across multiple roles, making them difficult for respondents to identify.”*
- While the broad range of backgrounds among data stewards is often viewed as a strength, enabling them to contribute effectively across a diverse research landscape the phrase *“fragmented professional identity*” and the following paragraph seems to imply a negative interpretation of the diverse educational backgrounds. You may want to consider rephrasing.
- It is also not clear that the data indicates that there is a *‘growing emphasis’ *on the technical dimension of the role given the sample size.
- We suggest the last paragraph in the conclusion is giving the heading/title **Next Steps **

Notes:

 There minor typos which need to be corrected and acronyms which should be clarified . Such as

 “ Consortium GARR”

 "a DS is a professional s with"

If applicable, is the statistical analysis and its interpretation appropriate?

Yes

Is the study design appropriate and is the work technically sound?

Yes

Is the work clearly and accurately presented and does it engage with the current literature?

Yes

Are the conclusions drawn adequately supported by the results?

Partly

Are sufficient details of methods and analysis provided to allow replication by others?

Yes

Are all the source data and materials underlying the results available?

Yes

Reviewer Expertise:

Research data stewardship

We confirm that we have read this submission and believe that we have an appropriate level of expertise to confirm that it is of an acceptable scientific standard.

# Response

**Published:** 2026-07-22

**Authors:** Giulia Caldoni

**Affiliations:** University of Bologna, Bologna, Emilia-Romagna, Italy

*We thank the referees for their positive feedbacks and for the thoughtful comments on our work. They allowed us to improve the text significantly, and to clarify some aspects that deserved more space and attention.* *Below, we provide a detailed point-by-point response to each comment. Our responses follow each of the reviewers’ comments.*

**Is the work clearly and accurately presented, and does it engage with the current literature?** The work is well-grounded in relevant literature and contextualised within the European research landscape. We encourage the authors revisit the statement *“only 33% of the European respondents actually had the job title “data steward” *The figure mentioned does not seem to be supported by the data underlying the cited report and potentially this needs to be rephrased for accuracy. Data available here https://zenodo.org/records/10117910

**Authors’ response:** We thank the reviewers for raising this point. We carefully re-examined the underlying data from the *Research Data Alliance Interest Group Professionalising Data Stewardship Career Tracks Survey Dataset* . Among the 205 European respondents who answered this question, 68 reported the job title *Data Steward*, whereas 137 reported other job titles. Thus, individuals with the job title *Data Steward* accounted for approximately 33% of the European respondents (68/205 = 33.2%). Based on this verification, we consider the statement in the Introduction to be accurate and supported by the underlying data. Therefore, no modification to the manuscript was deemed necessary.

**Is the study design appropriate and is the work technically sound?** The design is appropriate and socialised in relevant and available channels.

- The survey was reopened for a period could the reason and justification for this be included in the methods.
- Some additional details of the limitations of the survey would be useful such as an acknowledgement of the limitation of using mailing list which are open science focused so could be seen as self-selecting.

**Authors’ response: **We thank the reviewers for the helpful comments. To improve clarity, we revised the *Materials and methods* section to provide more details on the reasons for reopening the survey. We acknowledged and discussed potential limitations of the dissemination approach, including the survey being reopened, in the *Discussion* section.

- We also suggest including details of how well the 77 responses represent the full Italian research ecosystem – number of eligible institutions vs the number of responses and an estimated national research population.

**Authors’ response: ** We thank the referees for raising this point. To address this point, we revised the text to indicate that responses were obtained from 17 out of the 104 Italian universities and research centers. We explicitly discussed that this underrepresentation may affect the generalisation of the findings. We also clarified that the primary aim of the study is to provide a snapshot of current practices rather than a fully comprehensive or statistically representative national mapping. These additions can be found in the *Discussion* section.

- Could the authors also clarify how the list of data stewardship activities outline in Figure 7 was complied.

**Authors’ response: **We thank the reviewers for the suggestion. We have clarified the manuscript by specifying that the list of data stewardship activities presented in Figure 7 was developed through an iterative design process based on the authors’ professional experience and refined using insights from international reports, particularly those from the Netherlands and Denmark. This information has now been explicitly included in the Methods section.

**Are sufficient details of methods and analysis provided to allow replication by others?** The methodology is described in detail and is sound and appropriate. However, there could be some improvements.

- In figure 1 two of the data points beside the number 17 are the same for consistency should 2 be 1.5 times the size of 1. Pie charts for cities with 2-5 respondents were visualised as 1.5 the size of the units.
- Names of major cities could be included in figure 1 for those who are not familiar with the geography of Italy.
- Figure 8 – could the axis be explained perhaps include a legend to clarify what each point on the bar chart means. 

**Authors’ response:** We thank the reviewers for the suggestions. 

- We fine-tuned the pieces of information shown in figure 1 following referees' suggestions. First, we corrected the consistencies of the cities mentioned in the review, according to the logic mentioned in the Materials and Methods section of the manuscript.
- We decided to include labels for all cities to make it easier to identify the respondents' places of origin, even for those unfamiliar with the geography of Italy.
- We converted figure 8 into a table to improve the clarity of data interpretation.

**Are all the source data and materials underlying the results available?** Yes all data and material have been shared

- The underlying data provides the survey questions in English but could the column headers in the dataset also be translated to English.

**Authors’ response:** We thank the referees for the suggestion. Since the survey was conducted in Italian, the dataset was deposited in its original language to ensure fidelity to the source data. We believe that translating only the column headers, while leaving the response values in Italian, would reduce dataset readability and create ambiguity for data reuse. To support accessibility for non-Italian-speaking users, we have provided an English version of the survey questions in the accompanying documentation. For these reasons, we have retained the original Italian column headers in the archived dataset.  

**If applicable, is the statistical analysis and its interpretation appropriate?** The analysis is descriptive which is appropriate given the profile of the data and sample size.

- It may be helpful to refine some of the wording used in the descriptions of the findings. Softening the phrasing will ensure that the interpretations come across as balanced and precise. An example would be the phrase ‘striking majority’ which seems a bit of an overstatement.

**Authors’ response: ** We thank the reviewers for the observation. To improve the paper readability and the interpretation of the results, we carefully revised the manuscript to ensure the use of more cautious wording and to tone-down expressions that might have overemphasized the results. As an example, we removed the phrase ‘striking majority’.

**Are the conclusions drawn adequately supported by the results?** The authors acknowledge the limitations of the study in the conclusion, however there are several instances where the discussion appears to over-interpret or extend the findings beyond what can be supported by the data.  

- The conclusions section would benefit from being more concise and from reframing the content to highlight a small number of clear, well-defined take-home messages.  
- The conclusion section would also benefit from clarification of the authors definition of DS-like staff, DS- activities, DS-like responsibilities, DS-like professionals as they are mentioned in the discussion but not elsewhere. It would be good to under the meaning  of these phases in the context of the survey.

**Authors’ response: ** We thank the reviewers for the comments. We have carefully revised the *Discussion* section to make it more concise and ensure that interpretations remain consistent with the results. We have softened statements that could be perceived as overinterpretations and aligned the wording more closely with the exploratory scope of the study. We uniformed the terminology, using “DS functions” to what was referred as “DS-like task”, “DS-like responsibilities” and “DS-like activities” in the previous version. In the *Introduction section of the *revised text, we have clarified the terminology, where we explicitly state that the term “DS” “DS functions” and “data stewardship” are used in a broad sense to include professionals who perform data stewardship functions. We have also ensured consistent wording throughout the manuscript to avoid ambiguity.

- “In the future, we plan to perform another survey including as many universities and research institutions as possible.” We suggest removing this as it is should be an aim from the outset.

**Authors’ response: ** We thank the reviewers for the suggestion. We removed this specific sentence and revised the entire Discussions section highlighting the impact that the survey already had in the Italian and international landscape, rather than outlining future aims.

- We suggest that this statement is revisited “On the other hand, the large proportion of respondents who answered in a personal capacity may reflect limited institutional awareness/policies related to RDM needs or a lack of internal communication about existing practices.”  It is an over interpretation of the findings but could be rephrased as question.

**Authors’ response: ** We thank the reviewers for the observation. We revised the entire text to make it more adherent to the results we are presenting, hence we removed this sentence.

- For the following line it is not clear how this assertion  is supported by figure 6 and 7.  “According to the responses that we gathered here, a DS is a professionals with (at least one or more) cross-cutting skills in RDM (disciplinary, IT and technical, legal) and who very often acts as a bridge between researchers (i.e. producers and users of research data), infrastructures and research organisations (Figure 6 & Figure 7).”

**Authors’ response: ** We thank the reviewers for the remark, we removed this sentence as the same concept is better explained in a following paragraph of the *Discussion* section.

- The suggestion that the list of data stewardship task was ‘exhaustive’ seems to be an overreach considering the sample size and the early stage of data stewardship socialization and adoption in Italy in 2023 as described by the author themselves. Also in light of earlier statement such as “DS-like activities are performed informally or distributed across multiple roles, making them difficult for respondents to identify.”

**Authors’ response: ** We thank the reviewers for the observation. We revised the sentence, using a more cautious wording, and specified how the list of activities was compiled.

- While the broad range of backgrounds among data stewards is often viewed as a strength, enabling them to contribute effectively across a diverse research landscape the phrase “fragmented professional identity” and the following paragraph seems to imply a negative interpretation of the diverse educational backgrounds. You may want to consider rephrasing.
- It is also not clear that the data indicates that there is a ‘growing emphasis’ on the technical dimension of the role given the sample size.

**Author’s response: ** We thank the reviewers for the observation. We revised the sentences to make our statements clearer.

- We suggest the last paragraph in the conclusion is giving the heading/title Next Steps

**Authors’ response: ** Thank you for the suggestion. We revised the entire Discussions section to clarify our observations, hence we decided to highlight the impact that the survey already had in the Italian and international landscape, rather than to outline possible next steps. Notes: There minor typos which need to be corrected and acronyms which should be clarified . Such as “ Consortium GARR” "a DS s a professionals with"

**Authors’ response: ** We thank the reviewers for these notes, we carefully checked the text, corrected typos and explained acronyms. “GARR” can’t be explained as it is not an acronym. We provided a link to the Consortium GARR webpage for clarity.
